# The patient-doctor relationship questionnaire (PDRQ-9). An overview of 20 years of research and a proposal for normalisation of scores. Systematic review

**DOI:** 10.3389/frhs.2026.1754286

**Published:** 2026-02-19

**Authors:** Christina Maria Van Der Feltz-Cornelis, Edwin de Beurs

**Affiliations:** 1 Department of Health Sciences, University of York, York, United Kingdom; 2 Institute of Health Informatics, University College London, London, United Kingdom; 3 Clinical Psychology Department, University of Leiden, Leiden, Netherlands; 4 Department of Research, Arkin, Amsterdam, Netherlands

**Keywords:** patient doctor relationship questionnaire (PDRQ-9), scoring, standardization analysis, norms, validity, systematic review, patient doctor relationship

## Abstract

**Background:**

The Patient-Doctor Relationship Questionnaire (PDRQ-9) was developed as the first questionnaire to establish the quality of the Patient-Doctor Relationship (PDR) from the patient's perspective. It was published in 2004, has been translated and psychometrically tested in many languages, and is used widely.

**Objectives:**

This study aims to explore the reliability and validity of the PDRQ-9 in various languages and countries, to report norm scores and cut-off scores for an above or below-average relationship, and to propose a novel scoring method for the PDRQ-9 based on the findings.

**Methods:**

*Eligibility criteria:* studies should report on the PDRQ-9 item version for inclusion. Studies were excluded if they were not peer-reviewed, did not provide outcome data or analysis for the PDRQ-9, did not use the recommended scoring method for nine items, developed another questionnaire based on the PDRQ-9, were a protocol publication, or were retracted. *Information sources:* We searched for articles in Pubmed/Medline/Web of Science/Google Scholar with the terms “patient-doctor relationship questionnaire” OR “PDRQ-9” OR “PDRQ9” OR “PDQR” published between the publication date of the original study in March 2004 up to November 2025. No language restrictions were used. *Risk of bias:* was assessed by a tool for cross-sectional studies. *Synthesis of Results:* we present psychometric and factor structure findings and total scores, calculating weighted Means and SD__pooled_ over studies. We present an approach to convert raw sum scores to standardised, normalised scores.

**Results:**

The search resulted in 66 studies performed in primary care and various specialist general healthcare and mental healthcare settings in up to 24 countries. Twenty-five of those adapted the PDRQ-9 in 15 different languages and reported on its validity. Fourteen studies investigated the factor structure, and in all except one, the one-dimensional structure of the PDQR-9 was confirmed. Based on normalised *T*-scores, we recommend a cut-off value of ≤ 44 for a challenged PDR, 45 ≤ T ≤ 56 for an average PDR, and T > 56 for a good PDR.

**Discussion:**

Construct and criterion validity are well supported, with correlations between the PDRQ-9 score and patients reporting that they understood their illness well, experiencing more shared decision-making and adherence to treatment. *Limitations:* Most studies reported a ceiling effect in the scores with an overrepresentation of high ratings. The risk of bias was considered low to moderate. Most samples were convenience samples.

**Interpretation:**

The PDRQ-9 is an instrument that was psychometrically tested with its validity supported worldwide and fulfils a need. As the PDR is essential in health services, the PDRQ-9 is a highly relevant measure. It provides a good measure of the PDR when total scores are transformed to a metric with a normal distribution, yielding helpful information, especially when the PDR is challenged. Future studies should preferably report normalised sum scores over mean scores of the items, to improve interpretation in view of ceiling effects, and given the provided cut-off score levels, as well as for consistency in the international literature.

**Systematic Review Registration:**

https://www.researchregistry.com/browse-the-registry#registryofsystematicreviewsmeta-analyses/, reviewregistry1953.

## Introduction

The patient-doctor relationship (PDR) has been chiefly explored from the doctor's perspective, that is the primary care practitioner (PCP) or the medical specialist in the general hospital setting. Questionnaires to assess the patient's view on the patient-doctor relationship (PDR) were not available until 2004 when a study describing the development and validation of the Patient-Doctor Relationship Questionnaire (PDRQ-9) was published (1). It explores aspects of the patient-doctor relationship in 9 items. A description of the PDRQ-9 and proposed scoring method can be found in the Supplementary Materials.

This questionnaire was the first one developed to explore the PDR from the patient's perspective in a comprehensive way, as confirmed in a 2012 systematic review of instruments assessing the patient-doctor relationship. It explored aspects such as communication, the consultation process, and trust (2). A difference between the PDRQ-9 and patient satisfaction questionnaires was the decision not to focus on quality of care, technical items, or specific communication patterns. Instead, it focuses on the PCP's helping attitude as experienced by the patient, which was in line with the view that a helping alliance was at the core of a patient's relationship with their doctor. It was initially developed in Dutch, taking the Helping Alliance Questionnaire, that had been translated and psychometrically tested in Dutch, and assesses the therapeutic alliance in psychotherapy, as a starting point (3–5) and adapting it to address specific aspects relevant to a relationship between a patient and their medical doctor. This resulted in a short questionnaire containing nine questions: the PDRQ-9. The psychometric properties of the PDRQ-9 were assessed in collaboration with 110 general practice patients and 55 patients in an Epilepsy Clinic. The first publication on the Dutch PDRQ-9 provided an English translation of the questionnaire, including its instructions, items and response options. This English version was provided after forward and backward translation and discussion, independently, with two native English speakers. A study conducted in the USA, Canada and the UK, two other USA studies, and a study conducted in Nigeria reported on the validity of the English PDRQ-9. Two of those studies also performed confirmatory factor analysis that confirmed the one-factor structure of the English PDRQ-9, as shown in Table 1 (6, 7). This well-studied English version was the starting point for all translations in different languages.

**Table 1:** Factor structure findings of the PDRQ-9.

Study/Country	Study Population/Clinician	Factor structure indices
Van der Feltz-Cornelis et al. (1) The Netherlands	110 primary care patients in 5 Practices and 55 epilepsy inpatients in an specialised epilepsy clinic. Convenience sample. Primary care doctors and neurologists.	Principal component analysis with Varimax rotation of the 15-item version showed two factors. The Eigen value of the first factor was 7.509. This accounted for 58% explained variance. The Eigen value of the second factor was 1.198. This accounted for an additional 9%. The second factor and one item were eliminated, resulting in the nine item version PDRQ-9.
Porcerelli et al. (6) USA	180 adult primary care patients.	A principal components factor analysis with orthogonal varimax rotation was conducted. The results support a single factor solution with an eigenvalue of 7.04 accounting for 78.23% of the variance. No other component reached an eigenvalue of 1.0. Table 2 displays the distributions of each study variable. The mean values for men and women on both patient-rated and physician-rated variables did not differ statistically. Skewness and kurtosis values indicate that the study variables are relatively normally distributed.
Aloba et al. (7) Nigeria	309 outpatients with stable schizophrenia.	Confirmatory factor analysis confirmed the unidimensionality of the factor structure of the PDRQ-9. A one factor model of the PDRQ-9 had satisfactory goodness of fit indices on Confirmatory Factor Analysis (χ^2^/DF = 1.72; *p* = 0.030; GFI = 0.98; adjusted GFI = 0.95; comparative fit index = 0.99; Tucker–Lewis index = 0.98; standardized root mean square residual = 0.021; expected cross-validation index = 0.276; root mean square error of approximation = 0.048).
Martín-Fernández et al. (8) Spain	451 patients from six primary care centres in Madrid. Convenience sample. Primary Care Physicians.	Factor analysis found that a single factor explained 75.3% of the variance and showed very high loadings of all the items, except for the 2nd item.
Calderón et al. (9) This study used the translation of Martín-Fernández et al. (8) Spain	560 patients at 15 tertiary referral hospitals in Spain with colorectal (44.3%) or breast (34.3%) cancer. Convenience sample. Oncologists.	Confirmatory factor analysis was conducted. Given that the item scores were ordered-categorical, with strongly negative skewed distributions and the sample size was large, as most suitable factor-analysis procedure a non-linear item factor analysis model based on an underlying-variables approach was chosen, and a polychoric correlation matrix was used. The unidimensional model fitted the data well and the values of the standard indices displayed good fit in all cases: SRMS = .02; RMSEA = .05, and CFI = .99. Furthermore, the H value was.98, signifying that the factor was well defined by the 9 items and that the solution was strong and replicable. Finally, the ECV value was.98, indicating that 98% of the common variance of the item scores could be explained by the fitted single factor. So, the solution can be considered as essentially unidimensional. Thresholds and factor loadings could be constrained to be invariant across sex, age, and tumor site, indicating strong measurement invariance. Scores derived from the unidimensional structure exhibited satisfactory degrees of reliability and determinacy. The marginal reliability of the factor scores estimates based on the unidimensional factor-analytic solution exceeded.98 and the FDI value was.99. McDonald's omega reliability estimate based on the simple sum scores was.98, while the alpha estimate was.97.
Fernández Castillo et al. (10) Mexico	305 patients with T2DM presenting at a general hospital. Convenience sample, Endocrinologists.	A confirmatory factor analysis, using Maximum Likelihood method provided evidence of a good fit of the hypothesized one-factor model. All items presented an acceptable measurement weighs (*λ* > 0.5).
Zenger et al. (11) Germany	2,275 persons aged ≥14 who reported consulting with a primary care physician (PCP) in a representative cross-sectional German population survey. Representative sample. Primary care doctors.	Confirmatory factor analysis demonstrated that the hypothesized unidimensional structure of the PDRQ-9 fit the data very well [χ^2^ (27) = 345.860; χMIN/df = 12.810; GFI = .965; NFI = .979; CFI = .980; TLI = .974; SRMR = .019; RMSEA = .072]. The standardized regression coefficients of the latent variable “satisfaction with the patient-doctor relationship” varied between.72 and.88, indicating substantial relationships between the latent variable and each of the 9 items of the PDRQ-9.
Arafat (12) Bangladesh	50 participants older than 14 years of the Psychiatry Out-Patient Department (OPD) of Dhaka Medical College Hospital (DMCH). Convenience sample. Specialty mental health clinicians.	Construct validity was assessed by factor analysis. It showed high commonalities between the items before and after extraction ranging from 0.79 to 0.85. The varimax rotation showed only one component was extracted. The corrected item-total correlation was *r_it_* = ≥ 0.87 which was highly significant.
Wollman et al. (13) Brasil	133 adult users of a Primary Health Care Service in Porto Alegre, State of Rio Grande do Sul, Brazil. Convenience sample. Primary Care physicians.	Confirmatory factor analysis: All items presented factor loading > 0.5 in the factor analysis. The cross-cultural adaptation of the PDRQ-9 in Brazil replicated the factorial structure found in the original study, with high internal consistency.
Wang Y et al. (14) China	203 GH inpatients of neurology, gastroenterology, obstetrics and gynecology ward of Peking Union Medical College Hospital, Beijing. Convenience sample. Medical specialists.	Structural validity: The sample was randomly split in half to perform exploratory factor analysis (EFA) and confirmatory factor analysis (CFA), with IBM SPSS 20.0 and AMOS 27 respectively. Before the EFA was conducted, data suitability and sampling adequacy were checked using the Kaiser-Meyer-Olkin (KMO) value and Bartlett's test of sphericity. During the principal components analysis, factors with an eigenvalue larger than 1 were extracted. A total factor loading of more than 60% was considered as acceptable. Secondly, a confirmatory factor analysis [estimation method = diagonal weighted least square] was carried out. Exploratory Factor Analysis: The two-factor model of PDR quality and treatment quality was supported (χ^2^/*df* = 1.494, GFI = 0.925, RMSEA = 0.071, RMR = 0.008, CFI = 0.985, NFI = 0.958, NNFI = 0.980, TLI = 0.980, IFI = 0.986). According to the meaning and factor loading of each item, items 1−5 could be classified into a dimension named relationship quality, which describes the trust and empathy experienced by patients during the treatment. Items 6−9 were classified as another dimension, named treatment quality, which describes patients’ satisfaction with the process and results of his or her treatment received. The loading of each item on its corresponding factor was >0.7. IRT analysis: The discrimination parameters estimated ranged from 2.83 to 28.43. The MIRT model suggested a correlation of 0.93 between the two dimensions, with a 95% confidence interval of [0.89, 0.97], indicating that the two dimensions were highly correlated. So,although some support for two factors (relationship quality and treatment quality) was found, their high intercorrelation suggests that very similar construct are being measured.
Li et al. (15) China	505 outpatients of internet hospitals and physical hospitals in Wuhan. Convenience sample. Medical specialists.	χ^2^/df = 3.799, RMR = 0.020, RMSEA = 0.092, GFI = 0.935, CFI = 0.958, TLI = 0.944 and NFI = 0.949 in this study, which supports a one factor model.
Maleki et al. (16) Iran	208 patients in primary health centres in Tehran. Convenience sample. Primary Health Care Physicians.	Construct validity was evaluated with an Exploratory Factor and a Principle Component Analysis accompanied by a varimax rotation. One item loaded and one factor justified 68.01% of variance. With one factor, all items presented factor loading of >0.5. Eigenvalues of 6.12. A Confirmatory Factor Analysis used to evaluate the factor structure found the fit to be satisfactory. (PCFI = 0.626, AGFI = 0.923, CMIN/DF = 1.849, NFI=0.880, and RMSEA = 0.056)
Deniz et al. (17) Turkey	399 Patients from the province of Yalova, Turkey. Convenience sample (online). Primary care physicians.	Factor loadings range from 0.727 to 0.857. (PDRQ questions 1–5, 8: >0.8 PDRQ questions 6,7,9: >0.7)
Bener et al. (18) This study used the version of Deniz et al. (17) Turkey	1,031 inpatients from psychiatric clinics in Istanbul hospitals clinically diagnosed with schizophrenia (*n* = 141) bipolar disorder (*n* = 127), depression (*n* = 136), panic disorder (*n* = 79), phobia (*n* = 122), obsessive-compulsive disorder (*n* = 111), generalized anxiety disorder (*n* = 99), somatization (*n* = 119), post-traumatic disorder (*n* = 129), stress related disorders (*n* = 140), attention- deficit/ hyperactivity disorder (*n* = 33), dementia (*n* = 60).	The exploratory and confirmatory factor analyses confirmed validity and psychometric characteristics of the Turkish version of the 9-item Patient-Doctor Relationship Questionnaire (PDRQ-9) as follows. The corrected item-scale correlations ranged from *r* = .66 to.81. The scales items demonstrated satisfactory overall internal consistency (Cronbach's alpha = .89). The Exploratory and Confirmatory Factor Analysis (principal component analysis with Varimax rotation) with eigen value of 3.8, percentage of total variance explained 48.4%, Cronbach's alpha = .85, Kaiser-Meyer-Olkin Measure of Sampling Adequacy 0.904, and Bartlett's Test of Sphericity Chi- Square = 5,413.59 (*p* < 0.0001).

### Rationale

Additional research into the psychometric properties of the PDRQ-9 was recommended to investigate its reliability and validity in larger primary care or general hospital samples (1) and compare it to a measure assessing the patient-doctor relationship from the doctor's standpoint, such as the 10-item Difficult Doctor-Patient Relationship Questionnaire (DDPRQ-10) (19). Another recommendation was to explore whether the PDRQ-9 test scores correlate with clinically important outcomes such as higher clinical follow-up rates, patient compliance with treatment recommendations, illness outcomes, or functional status (1). Since its publication, this questionnaire has been adopted widely in the literature on the PDR. It was cited over 1,500 times in Google Scholar and has been translated, psychometrically tested and used globally for research-related evaluations. This abundance of publications on the PDRQ-9 enables us to explore some research questions regarding the abovementioned psychometric properties, and the cross-cultural validity of the PDRQ-9. Cross-cultural validity refers to whether measures that were originally generated in a single culture are applicable, meaningful, and thus equivalent in another culture (20). It has been mainly applied in psychological studies which need to adapt self-reported health status measures for use in languages other than the source language (21).

We aim to provide an overview of the developments with the PDQR-9 over the last 20 years, to summarise the findings, and to illustrate for what purpose the PDRQ-9 can be used. Furthermore, we propose a method for normalising scores and signposting areas of research that might be relevant to explore in the future.

### Objectives

1. Explore reliability and validity according to studies of the PDRQ-9 in different languages and countries.
2. Explore studies reporting norm and cut-off scores for an above or below-average PDR.
3. Propose a novel scoring method for the PDRQ-9 based on the findings.

## Methods

### Eligibility criteria

Studies presenting data on the PDRQ-9 that used 9-item versions of the PDRQ-9 and the scoring method of those items were eligible for inclusion. Studies were excluded if they were not peer-reviewed, did not provide outcome data for the PDRQ-9, did not use the recommended scoring method or 9 items, developed another questionnaire based on the PDRQ-9, were a protocol publication, or were retracted.

### Information sources

We conducted a search for articles in Pubmed, Medline, Web of Science, and Google Scholar, with the last date at the end of October 2025.

### Search strategy

We used the terms “patient-doctor relationship questionnaire” OR “PDRQ-9” OR “PDRQ9” OR “PDQR” (as some studies had reversed the letter order of the abbreviation) published between the publication date of the original study (1) in March 2004 up to November 2025. No language restrictions were used. We checked studies for references to find other studies fulfilling the inclusion criteria. In addition, we consulted a database of PDRQ-9-related studies that was put together over the years by one of the authors (CFC). The search string is mentioned at the end of the document.

### Selection process

Search and screening were conducted independently and in duplicate by the two authors (CFC or EdB) and an assistant, and in case of doubts, they were discussed until consensus was obtained. We outlined the selection of studies in a Flowchart.

### Data collection process

All performed data extraction, and in case of doubt, they discussed it until a consensus was reached. We queried 16 authors for additional data or clarification. Six authors (17, 22–26) responded and provided the requested information.

### Data items

We extracted data from the studies exploring countries and medical settings where the studies were conducted, the type of healthcare professionals who were evaluated with the PDRQ-9, patient populations in which the PDRQ-9 was administered, the method of language translation, if the translated version was provided, any validation or reliability outcomes, scoring method, and any outstanding issues relevant for further research mentioned in the publications. We also summarised findings based on the PDRQ-9 in studies where an already psychometrically tested version was used, like Mean, SD, total scores, and cut-off levels*.*

### Risk of bias

The authors (CFC, EdB) assessed the risk of bias using an adapted tool for cross-sectional studies (27), focusing primarily on selection (description target group), sampling method (representative or convenience sample), valid measurement of PDRQ-9 outcome, and selective reporting. Both authors discussed the criteria and assessed the studies in duplicate, examining cases of doubt together.

### Effect measures

M and SD, cut scores, norm scores.

### Synthesis methods

We present psychometric and factor structure findings and total scores, calculating weighted Means and SD__pooled_ over studies. We present an approach to convert raw sum scores to standardised, normalised scores. The most recent guideline on reporting transcultural validity states that it is not only important to consider semantically correct translation and conceptual equivalence. Testing of reliability and construct validity, psychometric analysis and field studies should also be conducted (28). We have structured the findings accordingly. Therefore, Table 2 reports on translation method, such as forward-backward translation (40) and psychometric findings, such as any reliability or validity findings; Table 1 reports on factor analysis, and Table 3 shows field studies with Means and SDs. Weighted mean item scores and SD__pooled_ are then reported in Table 4. Cut-off values for the quality of the patient-doctor relationship based on normalised *T*-scores are reported in Table 5. Mean scores are presented in Supplementary Table A1 of the Supplemental Materials. A crosswalk table and formulas for converting PDRQ-9 raw scores into normalised *T*-scores and Percentile scores is presented in Supplementary Table A2 in the Supplemental Materials.

**Table 2:** Psychometric studies of the PDRQ-9 reporting translation method, reliability or validity.

Study/Country	Study Population/Clinician	Language Translation method	Reliability (internal consistency, test-hertest, or other)	Any validity indices
Dutch version				
Van der Feltz-Cornelis et al. (1) Netherlands	110 primary care patients in 5 Practices and 55 epilepsy inpatients in an specialised epilepsy clinic. Convenience sample. Primary care doctors and neurologists.	The questionnaire was developed in Dutch and published in English after forward and backwards translation and independent evaluation by two native speakers. English version was provided.	Internal consistency: Cronbach's *α* = .94. Test-retest reliability as assessed with Pearson correlations was *r* = .61, and significant.	Construct – discriminant validity was supported: The mean PDRQ-9 score was 4.06 (SD.77) in the primary care setting and 3.60 (SD 1.09) in the epilepsy clinic, a significant difference (mean.46, p.001).
Hoytema van Konijnenburg et al. (29) Netherlands	82 parents who underwent the “Amsterdam Protocol” at an outpatient paediatric department. Convenience sample. Pediatric staff.	The primarily used Dutch version (1) was used for this study.	Internal consistency: Cronbach's α = .89	
Afaan Oromo Version				
Biyazin et al. (30) Ethiopia	372 surgical patients at Jimma Medical Center. Systematic Random Sampling method. Surgical healthcare providers.	Forwards-backwards translation method. Translated version was not provided.	Internal consistency: Cronbach's α = .86	
Arabic version				
Hegazy et al. (31) Egypt	434 Patients in outpatient family health clinics in the Suez Governorate, Egypt. Convenience sample. Primary care physicians.	“The adaptation to Arabic was performed according to the state-of-the-art procedure of forward-backward translation by two medical doctors and one English-Arabic bilingual translator. Two forward translations into Arabic were independently completed by 2 medical doctors, both native speakers of the Arabic language and fluent in English. The 2 Arabic versions were compared, and an updated Arabic forward version was compiled. This version was translated back into English by a professional translator with experience in medical translation. This translator had not been involved in the forward translation. The primarily published version and the back-translated version – both in English – were compared by the medical doctor and the expert translator. Thus, an optimized Arabic version was generated. Additionally, in a final reconciliation process, the final Arabic version (PDRQ-9 Arabic) was generated and approved by all parties. Preliminary changes to the original questionnaire were made after piloting the tools and were reviewed.” Translated version was not provided.	Internal consistency: Cronbach's α > .979.	
Bangla/Bengali version				
Arafat (12) Bangladesh	50 participants older than 14 years of the Psychiatry Out-Patient Department (OPD) of Dhaka Medical College Hospital (DMCH). Convenience sample. Specialty mental health clinicians.	Bengali version Forward-backward translation Translated version was not provided.	Internal consistency: Cronbach's α = .97.	
Chinese version				
Wang Y et al. (14) China	203 GH inpatients of neurology, gastroenterology, obstetrics and gynecology, ward of Peking Union Medical College Hospital, Beijing. Convenience sample. Medical specialists.	“The questionnaire was translated using a forward-backward translation method including steps of initial translation, synthesis of the translations, back translation, expert committee review, and test of the prefinal version. Five psychiatrists first translated the questionnaire into the Chinese version from the English version and then synthesized into the initial version. Next, this version was back translated into English by a bilingual expert, and then compared with the original English version to identify any discrepancy in meaning. After the expert committee reached an agreement on the translated Chinese version, this version was used for pretest data collection. Nineteen patients recruited from the neurology ward took part in the pretest, and agreed that there was no discrepancy in meaning, thus forming the final version of the scale.” Translated version was not provided.	Reliability analysis: Cronbach's a was used to evaluate the internal consistency of the Chinese version of PDRQ-9 and its subscales. The Pearson correlation coefficient between the first test and the retest was calculated to access the 7-day test-retest reliability. Internal consistency: Cronbach's α = .865–.933. The 7-day test-retest reliability of the scale was.730.	Construct validity: Convergent validity and divergent validity: The Pearson correlation coefficients between patients’ age, PHQ-9 rating, as well as total score of PDRQ-9 and its subscales were calculated. After the correlation analyses were conducted, all the participants were divided into two groups according to their ratings on PHQ-9. Patients who scored 10 or above were identified with significant depressive symptoms. ANCOVA adjusted for age was conducted to verify whether there was statistical difference in PDRQ-9 ratings between participants with or without significant depressive symptoms. The PDRQ-9 and both subscales showed significant correlation with PHQ-9 (*r* = −0.196 to −0.309). ANCOVA analysis adjusted for age revealed significant difference in PDRQ-9 ratings between patients with or without significant depressive symptoms (*P* = 0.019).
Li et al. (15) China	505 outpatients of internet hospitals and physical hospitals in Wuhan. Convenience sample. Medical specialists.	Forward-backward translation. “All the items were translated from English to Chinese for the PDRQ-9 by an expert proficient in both Chinese and English, further discussed and approved by a focus group based on their expertise, and finally back-translated from Chinese to English by another qualified translator. A presurvey was performed to validate the developed questionnaire in 67 patients (14 users of internet hospitals). Context-specific adjustments were then made according to the feedback from the pilot survey.” Translated version was not provided.	Internal consistency: Cronbach's α = .921.	.
English version				
Egeli & MacMillan (32) USA, Canada, UK	190 patients with Fibromyalgia Online (USA, Canada, Uk) Convenience sample	The primarily published English version (1) was used for this study.	Internal consistency: Cronbach's α = .97.	
Porcerelli et al. (6) Detroit, USA	180 adult primary care patients. Convenience sample. Primary care faculty physicians and residents.	The primarily published English version (1) was used for this study.	Internal consistency: Cronbach's α = .96.	Construct validity: PDRQ-9 ratings were higher from patients who were treated by faculty physicians than from those who were treated by residents (*p* = .01). The PDRQ-9 is correlated negatively with the Difficult Doctor Patient Relationship Questionnaire (DDPRQ-10) (*r* = −.22, *p* = .003) but was not significantly correlated with patient age, health, or psychological distress.
Aloba et al. (7) Nigeria	309 outpatients with stable schizophrenia.	The primarily published English version (1) was used for this study.	Internal consistency: Cronbach's α = .92. Test-retest reliability was also satisfactory [Intra-class correlation coefficient *r* = 0.74 (0.65, 0.80), *p* < 0.001], indicating modest test-retest reliability after two weeks.	Construct validity: was supported by modest positive correlations with the Trust in physician scale (*r_p_* = 0.413, *p* < 0.001), duration of treatment (*r_p_* = 0.114, *p* = 0.044), number of previous hospitalizations (*r_p_* = 0.179, *p* = 0.002), and negative correlation with the Morisky Adherence Scale (*r_p_* = 0.174, *p* = 0.002).
German version				
Zenger et al. (11) Germany	2,275 persons aged ≥14 who reported consulting with a primary care physician (PCP) in a representative cross-sectional German population survey. Representative sample. Primary care doctors.	German Forward-backward translation of the English version Translated version was provided.	Internal consistency: Cronbach's α of the total score = .95. All corrected item-total correlations were ≥.94.	Construct validity was supported: The Spearman rank correlation between the mean satisfaction index and the satisfaction with pain treatment of 489 participants who reported chronic pain and pain treatment was *r* = .51. This result demonstrates acceptable convergent validity for this subsample. In an ANOVA that adjusted for age, the mean satisfaction index of participants with a potential depressive disorder (*N* = 218) was 3.66 (SD = .86), and that of participants without a potential depressive disorder (*N* = 2,030) was 4.12 (SD = .66) (F = 65.8, *p* = .001). Potential depressive disorder primarily accounted for a group difference in mean satisfaction index (F = 119, *p* = .0001), with a small effect size (Partial g^2^ = .05). The partial g2 of age was .007 (F = 7.1, *p* = .001). This result demonstrates acceptable discriminative concurrent criterion validity.
Hebrew version				
Zolotov et al. (33) Israel	Ninety-five patients who were prescribed medical cannabis for cancer, chronic pain or other and received a licence from the Medical Cannabis Unit of the Israeli Ministry of Health (MOH). Convenience sample. Medical cannabis prescribing specialists.	Hebrew version. Forward-backward translation and adaptation to reflect PDR in case of use of medical cannabis. Translated version was not provided.	Internal consistency: Cronbach's α = .803.	Construct validity: With demographic variables controlled, PDRQ-9 score was found to be significantly predicting adherence (*p* < 0.01).
Malay version				
Johny et al. (34) Malaysia	906 Patients who have reported using traditional and complementary medicine in Sarawak, Malaysia Convenience sample. Primary care physicians	Backward translation method with a pilot study for validity.	Internal consistency: Cronbach's α ranged from.617 to.948, indicating adequate internal consistency and reliability.	Content validity: Two biostatisticians expert in the field examined the content validity of the instrument.
Maldivian version				
Hassan et al. (35) Maldives	172 patients with essential hypertension. Maldives. Convenience sample. Primary care physicians.	Forward-backward translation process. Translated version was not provided.	Internal consistency: Cronbach's α = .89 in a pilot study with 30 patients.	Content validity: Face validity, by subjective assessment of whether the questionnaire *appeared* to measure what it was supposed to.
Persian Version				
Maleki et al. (16) Iran	208 patients in primary health centres in Tehran. Convenience sample. Primary Health Care Physicians.	Forward-backward translation of the English version. Trained assistant for illiterate patients.	Internal consistency: Cronbach's α ≥ .94.	Content validity: Face validity had an acceptable impact score. Each question had >1.5 impact value. Six experts evaluated content validity, and each question had a score of 1 in the content validity ratio. This used a three-part spectrum. An acceptable score was obtained based on the Lawshe tab (99% and >99%).
Brazilian (Portuguese) version				
Wollman et al. (13) Brasil	133 adult users of a Primary Health Care Service in Porto Alegre, State of Rio Grande do Sul, Brazil. Convenience sample. Primary Care physicians.	Portuguese version. Forward-backward translation. Translated version was not provided. Self-report and interview version (for analphabetic people).	Internal consistency: Cronbach's α = .94 in the self-report version.	
Spanish version				
Martín-Fernández et al. (8) Spain	451 patients from six primary care centres in Madrid. Convenience sample. Primary Care Physicians.	Spanish 9 item version, based on Mingote 15 item translation (36): The Patient-Doctor Relationship Questionnaire was adapted to Spanish. The questionnaire was translated into Spanish and then a back translation was made into English, evaluating the level of accuracy and adaptation of the differences found. Translated version was not provided.	Internal consistency: Cronbach's α = .952. The PDRQ-9 questionnaire shows a high internal consistency.	Construct validity was supported: Age (OR 1.03, 95% CI: 1.02–1.05) was associated with above average PDR index. Primary care users feel their relationship with their family physicians are very satisfactory, particularly in those who are older.
Calderón et al. (9) Spain	560 patients at 15 tertiary referral hospitals in Spain with colorectal (44.3%) or breast (34.3%) cancer. Convenience sample. Oncologists.	Spanish The psychometrically tested Spanish version (8) was used for this study. Translated version was not provided.	Internal consistency: Cronbach's α = .97.	Construct validity was supported: Evidence of convergent validity was supported by modest positive correlations with functional (*p* < .001) and global quality-of-life (*p* < .001) and negative correlations with psychological distress (*p* < .001). Low PDR index with the oncologist was associated with anxiety (*p* = .006), and depression (*p* = .004).
Fernández Castillo et al. (10) Mexico	305 patients with T2DM presenting at a general hospital. Convenience sample, Endocrinologists.	Spanish – Mexican version. Translated version was not provided.	Internal consistency: Cronbach's α = .95.	Construct validity was supported: “This study suggests that this instrument is a valid instrument useful for measuring doctor-patient relationship in patients diagnosed with Type 2 Diabetes Mellitus.”
Eiroa-Orosa et al. (37) Spain	1,562 patients in three primary care centres with gastroenterology services in Barcelona. Convenience sample.	The psychometrically tested Spanish version (8) was used for this study.	Internal consistency: Cronbach's α = .946	
Thai version				
Pitanupong et al. (38) Thailand	264 University Hospital psychiatry outpatients with depression. Convenience sample. Psychiatrists.	Forward translation from the original English version to the Thai version.	Internal consistency: Cronbach's α = .95.	Content validity of the translated version was evaluated by five psychiatrists, resulting in a CVI score of 0.8.
Praha et al. (39) Thailand	159 patients with Continuous ambulatory peritoneal dialysis in Thailand. Convenience sample.	The psychometrically tested Thai version (38) was used for this study. Translated version was not provided.	Internal consistency: Cronbach's α = .95.	
Sangngam et al. (24) Thailand	240 patients with asthma visiting tertiary hospitals in Thailand. Convenience sample.	Back-translation from the original English questionnaire.	Internal consistency: Cronbach's α = .93	
Turkish version				
Deniz et al. (17) Turkey	399 Patients from the province of Yalova, Turkey. Convenience sample (online). Primary care physicians.	The PDRQ-9 scale that was developed in the original study (1) was translated into Turkish. Expressions were made suitable for the study. “In Turkish, the words “doctor” and “physician” are used in the same sense. In this study the word “doctor” was used because “doctor” is more common and understandable in Turkish.”	Internal consistency: Cronbach's α = .932	Construct validity: “We analyzed the collected data with Smart PLS3 software and performed validity studies and other analyses.”

**Table 3:** Field studies reporting total scores (*N* = 25,865).

Study	Patient setting	N	M	SD
*Dutch*		***957***		
Van der Feltz-Cornelis et al. (1) The Netherlands	Primary Care patients	110	36.54	6.93
	Epilepsy clinic inpatients	55	32.40	9.81
Van der Feltz-Cornelis et al. (41) The Netherlands	Primary Care patients with somatoform disorders	81	27.67	5.98
Du Long et al. (42) The Netherlands	Thirty-three orthopaedic surgeons and 172 patients with either knee or hip osteoarthritis in Slotervaart Hospital, Amsterdam.	172	Not reported	Not reported
Hoytema van Konijnenburg et al. (29) The Netherlands	Parents who underwent the “Amsterdam Protocol” at an outpatient paediatric department.	82	38.88	6.12
Metz et al. (43) The Netherlands	SDM	94	28.35	10.35
	Control	106	27.18	11.88
Hanssen et al. (44) The Netherlands	Primary care patients >60 years with chronic MUS	107	36.84	7.98
	Primary care patients >60 years with chronic medically explained symptoms	150	39.86	7.14
		*N*	*Weighted M*	*SD_pooled_*
		*785*	*31*.*15*	*8*.*45*
*English*		***1,920***		
Georgopoulou et al. (45) UK	Hospital outpatients with lupus nephritis.	98	36.00	8.19
Porcerelli et al. (6) Detroit, USA	Visiting patients from a suburban university-based primary care clinic.	180	40.67	6.64
Aloba et al. (7) Nigeria	Specialty mental health outpatients with stable schizophrenia.	309	32.40	7.09
Hageman et al. (46) USA	Orthopaedic Hand and Upper Extremity Service hospital outpatients with carpal tunnel syndrome	84	37.80	7.83
Hageman et al. (47) USA	Orthopaedic Hand and Upper Extremity Service hospital outpatients with trigger finger	84	39.60	6.03
Gonzalez et al. (22) USA	Patients from five orthopaedic specialist offices in urban areas.	102	40.83	7.35
Magidson et al. (48) USA	Patients undergoing HIV treatment in substance abuse centre.	77	39.16	7.43
Kirby et al. (49) USA English version adapted for use with this population	Parent child pairs, child age 1–21 years, in urban primary care practice in Baltimore. First application of PDRQ-9 in pediatric patients/ children.	218	39.8	7.30
Alomran et al. (50) Saudi Arabia (English version)	Type 2 diabetes patients attending primary health-care centers of Al-Ahsa region in Saudi Arabia.	366	Not reported	Not reported
Din et al. (51) Lahore, India (English version)	Surgical and physiotherapy outpatients of 5 orthopedic hospitals (148)			
	Orthopedic surg	73	29.08	4.21
	Physiotherapists	75	35.61	4.61
Versluijs et al. (52) Texas, USA.	Initial musculoskeletal specialist surgery outpatient evaluations	34	Not reported	Not reported
Perche et al. (53) USA	Subjects with a clinical diagnosis of rosacea in a dermatology clinic.	30	Not reported	Not reported
Egeli & MacMillan (32) Online (USA, Canada, UK)	Patients with Fibromyalgia	190	24.22	10.66
		*N*	*Weighted M*	*SD_pooled_*
		*1,490*	*34*.*91*	*7*.*52*
*Spanish*		***3,677***		
Martín-Fernández et al. (8) Spain	Patients from six primary care centres in Madrid.	451	39.69	7.31
Calderón et al. (9) The Spanish PDRQ-9 psychometrically tested by Martín-Fernández et al. (8) was used Spain	560 patients at 15 tertiary referral hospitals in Spain with colorectal (44.3%) or breast (34.3%) cancer.	560	42.0	5.10
Eiroa-Orosa et al. (37) The Spanish PDRQ-9 psychometrically tested by Martín-Fernández et al. (8) was used Spain	Patients in primary care centres with gastroenterology services with congruent views with their physician	1,002	41.84	4.25
	Patients with incongruent views with their physician	560	41.91	4.21
Sarabia-Tapia et al. (54) The Spanish PDRQ-9 psychometrically tested by Martín-Fernández et al. (8) was used Mexico	Patients with Parkinsons disease in neurological hospital clinic.	100	26.10	8.70
Pascual – Ramos et al. (23) Mexico The Spanish PDRQ-9 psychometrically tested by Martín-Fernández et al. (8) was used	Outpatients with rheumatic diseases of a rheumatological clinic.	600	37.34	8.4
Peña-Valenzuela et al. (55) Mexico	Patients with systemic arterial hypertension (SAH) from a family practice in Mexico.	289	Not reported	Not reported
Torres-Reyes et al. (56) Mexico Mexican Spanish translation by Fernández Castillo et al. (10) was used	Primary care setting	115	Not reported	Not reported
		*N*	Weighted *M*	SD_pooled_
		*2,788*	*40*.*94*	5.28
*Italian*		***545***		
Montanaro et al. (57) Italy	Patients of the Neuroscience dept. of University of Torino.	24	37.30	7.30
Buizza et al. (58) Italy	Patients with breast cancer in three oncology clinics in Northern Italy.	164 (Question Prompt sheet) 160 (Question List)	42.67 42.64	4.10 4.20
Lauricella et al. (59)	Patients with neuroendocrine tumors during Wave 1 of the COVID pandemic	197	39.24 W1 39.15 W2	6.84 5.04
		*N*	*Weighted M*	*SD_pooled_*
		*545*	*42*.*29*	*4*.*43*
*Romanian*		***55***		
Stefanescu et al. (60) Romania	Children and adolescents from Romania with Type 1 diabetes.	55	18.96 (pre ACT intervention) 33.02 (post ACT intervention)	10.34 5.74
*German*		***2,542***		
Zenger et al. (11) Dinkel et al. (61) The German version of Zenger et al. (11) was used Dinkel et al. (62) The German version of Zenger et al. (11) was used Schmalbach et al. (63) The German version of Zenger et al. (11) was used Germany	A representative sample of the German population was selected with the assistance of a demographic consulting company	2,266 2,275	37.08	6.30
Hefner et al. (64) The German version of Zenger et al. (11) was used Germany	Patients treated with capecitabine at the Comprehensive Cancer Center of the University of Wuerzburg, Germany.	58	39.92	5.97
Hefner et al. (65) The German version of Zenger et al. (11) was used Germany	Cancer patients undergoing chemotherapy with capecitabine at outpatient clinics, day hospitals and doctors’ offices of the Comprehensive Cancer Center of the University of Wuerzburg, Germany.	64	39.87	5.76
Engler et al. (66) The German version of Zenger et al. (11) was used Germany	7 practices participating in a quality of care improving project named “Cardiovascular offensive”, Luebeck.	54	41.85	3.87
Markovic et al. (67) The German version of Zenger et al. (11) was used Austria	A cross-sectional study in TNB individuals, 18 or older, residing in Austria, and previously or currently undergoing medical transition, of whom 33.0% and 25.3% self-identified as trans men and trans women, respectively, and 41.8% as non-binary.	91	33.55	8.83
		*N*	*Weighted M*	*SD_pooled_*
		*2,542*	*37*.*19*	*6*.*35*
*Hebrew*		***93***		
Zolotov et al. (33)	Adherent patients prescribed medical cannabis for cancer, chronic pain/other	76	35.70	3.60
	Nonadherent	17	30.30	4.60
		*N*	*Weighted M*	*SD_pooled_*
		*93*	*34*.*71*	*3*.*79*
*Arab*		***1,053***		
Alghabiwi et al. (68) Saudi Arabia	Female primary care patients with chronic disease.	253	25.92	5.84
Al-Noumani et al. (69) The Arabic version of Hegazy et al. (31) was used Oman	Participants were recruited from outpatient specialty clinics across ten governorates in Oman.	800	33.14	7.55
		*N*	*Weighted M*	*SD_pooled_*
		*1,053*	*31*.*41*	*7*.*18*
*Bangla (Bengali)*		***264***		
Arafat et al. (12)	Participants older than 14 years of the Psychiatry Out-Patient Department (OPD) of Dhaka Medical College Hospital (DMCH).	50	28.50	10.20
Arafat et al. (70) Bangladesh	Outpatients of Dhaka Medical College and some private practices in the city of Dhaka.	214	38.43	8.95
		*N*	*Weighted M*	*SD_pooled_*
		*264*	*36*.*55*	*9*.*20*
*Brazilian (Portuguese)*		***6,921***		
Wollman et al. (13)	Adult users of a Primary Health Care Service in Porto Alegre, State of Rio Grande do Sul, Brazil.	133 133 623	40.05 39.87 29.07	6.30 6.30 7.20
Wollmann et al. (71)	Primary care sample in Brasil.	6,160	29.79	7.29
		*N*	*Weighted M*	*SD_pooled_*
		*6,921*	*29*.*92*	*7*.*26*
*Thai*		***1,205***		
Pitanupong et al. (38) Thailand	University Hospital patients.	264	39.20	5.30
Pitanupong et al. (72) Thailand	Patients in 4 psychiatric outpatient clinics.	542		
Praha et al. (39) The Thai version of Pitanupong et al. (38) was used Thailand	Patients with Continuous ambulatory peritoneal dialysis in Thailand. Convenience sample.	159	37.90	4.30
Sangngam et al. (24) The Thai version of Pitanupong et al. (38) was used Thailand	Patients with asthma. Convenience sample.	240	37.36	6.45
		*N*	*Weighted M*	*SD_pooled_*
		663	38.22	5.54
*Chinese*		***4,555***		
Li et al. (15) China	Outpatients of internet hospitals and physical hospitals in Wuhan (*N* = 505).			
	Internet hospital users	130	32.71	5.72
	Regular hospital users	375	34.17	4.85
Liang et al. (73) Taiwan	Older veterans	256	34.30	5.80
Qiao et al. (74) Inner Mongolia Autonomous Region of People's Republic of China	Patients from one provincial and one city-level general public hospital in Hohhot city, the capital of Inner Mongolia Autonomous Region of People's Republic of China	713	31.36	7.56
Zhou et al. (75) China	Online survey participants in the general population who visited a doctor in the last year. Convenience sample. (*N* = 1,903)			
	Before COVID-19 pandemic.	1,903	34.74	6.03
	During the COVID-19 pandemic		37,62	4.59
Wu et al. (26) China This PDRQ-9 version was translated and back-translated from the German version (11) into Mandarin Chinese. A one-factor structure was confirmed although not reported in the publication. (Written information by author (25)	Chinese general hospitals outpatients			
	High somatic symptom severity	248	38.03	6.25
	Low somatic symptom severity	236	37.01	6.91
Xiong et al. (25) China This PDRQ-9 version was translated and back-translated from the German version (11) into Mandarin Chinese. A one-factor structure was confirmed although not reported in the publication. (Written information by author (25)	Outpatients from ten outpatient departments at Chinese general hospitals (*N* = 491).			
	Low distress	202	38.8	5.70
	Somatically distressed	89	38.8	5.70
	Psychologically distressed	51	35.1	7.20
	Mixed distressed	149	36.0	7.20
Wang et al. (14) China	General Hospital inpatients of neurology, gastroenterology, obstetrics and gynaecology ward of Peking Union Medical College Hospital, Beijing.	203	33.45	4.44
		*N*	*Weighted M*	*SD_pooled_*
		*4,555*	*34*.*63*	*6*.*19*
*Persian*		***683***		
Maleki et al. (16) Iran	Patients in primary health centres in Tehran.	208	32.49	9.90
Torabipour et al. (76) Iran	Patients in community health centres in Ahvaz city	200	28.50	7.91
Ghorrabi et al. (77) Iran	Cardiology patients		35.17	7.84
	Gynecology patients		34.39	4.62
	Ophthalmology patients		36.29	6.03
	ENT patients		33.00	3.31
	Orthopedic patients		31.17	7.52
	Internal patients		34.30	7.15
	Surgical patients		32.17	7.93
	Dermatology patients		34.35	6.38
	Patients in other specialised clinics		33.81	6.98
	Total group	275	33.92	6.80
		*N*	*Weighted M*	*SD_pooled_*
		*683*	*31*.*90*	*8*.*17*
*Malay*		***906***		
Johny et al. (34) Malaysia	Patients who used traditional and complementary medicine	906	30.60	5.99
*Afaan Oromo*		***372***		
Biyazin et al. (30) Ethiopia	Surgical patients at Jimma Medical Center.	372		
*Maldivian*		***172***		
Hassan et al. (35) Maldives	Patients with essential hypertension	172	Not provided	Not provided

**Table 4:** Weigthed mean item scores (and SD_pooled_) and response frequencies.

Item	M_weighted_	SD_pooled_	% Not at all	% Somewhat	% Appropriate	% Mostly	% Totally
Item 1	3.75	0.91	2.4	6.8	44.1	23.4	23.2
Item 2	3.47	1.02	7.0	11.4	41.5	21.0	19.0
Item 3	3.77	0.93	2.7	6.7	41.1	24.7	25.2
Item 4	3.67	0.92	2.6	6.5	43.9	23.8	23.2
Item 5	3.70	0.93	3.0	6.8	42.3	23.0	24.8
Item 6	3.63	0.93	3.4	6.9	46.6	21.3	21.9
Item 7	3.71	0.93	2.6	6.0	44.7	22.7	24.0
Item 8	3.74	0.88	2.4	5.9	41.1	24.4	26.1
Item 9	3.54	0.97	6.0	10.0	40.9	20.3	22.6

**Table 5:** Cut-off values for quality of the patient-doctor relationship based on normalized *T*-scores.

Raw score	*T*-score	Interpretation
RS < 23	T < 30	Severely challenged
23 ≤ RS ≤ 30	30 ≤ T ≤ 39	Moderately challenged
31 ≤ RS ≤ 34	40 ≤ T ≤ 44	Mildly challenged
35 ≤ RS ≤ 42	45≤ T ≤ 56	Average
42 < RS	56 < T	Good

### Data preparation

Some authors sum the item responses to obtain a total score on the PDQR-9, while others calculate the mean score of the items. To present scores in the tables on the same metric, we multiplied the latter mean scores and SDs by 9 to obtain a total score. We calculated weighted Mean Scores and SD__pooled_ as an estimate of the SD in the total group, taking the N of the study into account, and produced a forest plot per study, per language group, and for the total group. As the scores on the PDRQ-9 appeared skewed, with most patients rating their doctors positively, we presented an approach to convert raw sum scores to standardised, normalised scores based on a large German representative sample dataset of the general population (11).

### Reporting bias assessment

We report the risk of bias in Supplementary Table A3 in the Supplemental Materials.

### Certainty assessment

We asked the authors of the three potentially representative samples for an anonymised subsample of their dataset to explore the possibility of converting raw sum scores to normalised scores and received two samples: the German (11) and the Brazilian (Portuguese) (71) one. The German sample was the only representative general population sample and was used to establish a conversion table and formula, as described below.

### Norms for the PDRQ-9 with normalised *T*-scores

PDRQ-9 total scores were first normalised with a percentile rank order-based approach [Rankit normalisation (18, 78, 79) with the RankNorm function in the R RNOmni package version 1.0.1 (80)]. This function first transforms raw scores to percentile ranks, based on the formula PR = (CF -.5*F)/N (CF = the cumulative frequency of the raw score; F = - the frequency of the Raw score; *N* = the size of the normative sample). Next, the percentile rank order scores were converted to standard scores (*Z*-scores) with the probit function, resulting in *Z*-scores with M = 1 and SD = 1). These *Z*-scores were converted to *T*-scores with T = 10*Z + 50 to obtain a more convenient metric with a M = 50 and SD = 10 (81). According to the empirical rule for the normal distribution, 68% of the population will score in the range of T = 40–60, 95% in the range of T = 30–70, and 99.7% in the range of 20–80. Once the T scores have been established, the curvilinear relation between raw scores and normalized *T*-scores can be estimated with non-linear modelling (the Non-linear Least Squares nls function in the R Stats (version 3.6.2) package and the glsnls package version 1.2.0 (https://cran.r-project.org/web/packages/gslnls/gslnls.pdf). The full procedure is described elsewhere (82). A crosswalk table and formula were established to convert raw PDRQ-9 scores to normalised T scores. We followed the Prisma statement guidelines for systematic reviews and report the PRISMA checklist in the Supplementary Materials (83).

## Results

**Figure 1:**
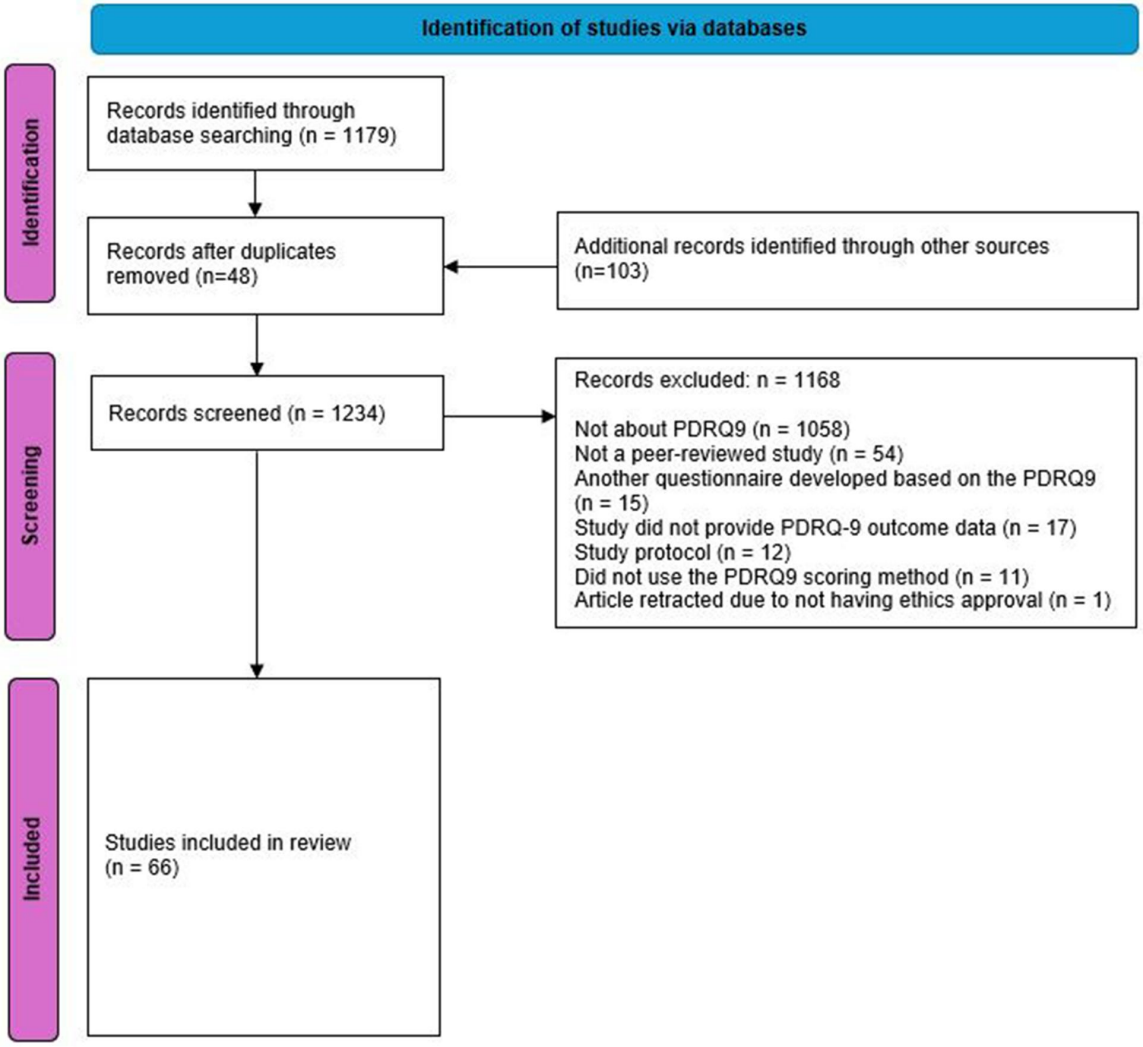
PRISMA 2020 flow diagram for systematic reviews.

The search resulted in 1,179 studies. Another 103 studies were identified via alternative searches, totalling 1,282 studies that used questionnaires and discussed the PDR. After 48 duplicates were removed, 1,281 studies remained. After screening, a total of 1,168 studies were excluded. 1,058 were excluded as they discussed the PDR but did not report on the PDRQ-9. Fifty-four not peer-reviewed studies were excluded. Fifteen studies (36, 84–97) developed a different questionnaire based on the PDRQ-9. Seventeen studies (2, 98–112) did not or insufficiently provide data on the PDRQ-9. Twelve articles were a study protocol (43, 113–122). Eleven studies (123–133) used a scoring method different from the recommended scoring method for the PDRQ-9. Finally, one publication was retracted due to not having ethics approval for the study (134). This resulted in 66 studies as indicated in the Flowchart (Figure 1).

Table 2 provides details of the 25 studies that adapted the PDRQ-9 in 15 different languages and collected psychometric information from 10,321 patients in a range of healthcare settings. The studies reported predominantly on construct and content validity. Reliability was mostly explored by Cronbach's alpha.

### Healthcare settings and patient populations

Regarding settings and patient populations, the psychometric studies were conducted with 4,555 primary care patients (1, 6, 8, 11, 13, 16, 17, 31, 34, 35, 37); *n* = 2,275 of these were from a representative general population sample (11). Eight studies were conducted in 2,338 general hospital outpatients; one in patients with cancer (9), one in patients with cancer or chronic pain receiving medical cannabis (33); one in patients with type 2 Diabetes Mellitus (10); one in patients of internet hospitals (15), one in surgical patients (30); one in orthopaedic patients (22); one in patients with Continuous Ambulatory Peritoneal Dialysis (39) and one in patients with asthma (24). Three studies were conducted with 623 outpatients of specialised mental health institutions (12), one of them specifically for patients with schizophrenia (7) and one for patients with depression (38). Two studies reported on 258 general hospital inpatients: one specifically on neurology, gastroenterology, obstetrics and gynaecology inpatients (14), and one on inpatients in a specialised epilepsy clinic (1). Furthermore, psychometric properties of the PDRQ-9 were evaluated in 82 parents of children presented at a paediatric clinic (29) and in 190 online patients in the general population with fibromyalgia (32).

### Construct validity – treatment settings

Construct validity was evaluated by comparing the PDRQ-9 score in different treatment settings. Primary care patients scored significantly better than epilepsy inpatients of a specialised epilepsy clinic (mean difference = .46, *p* = .001) (1). Primary care patients who were treated by faculty physicians scored higher than those who were treated by resident family doctors (*p* = .01) (6). There was a correlation between the PDRQ-9 score and duration of treatment (r*_pmcc_* = 0.114, *p* = 0.044) and number of previous hospitalizations (*r_pmcc_* = 0.179, *p* = 0.002), and a negative correlation with the Morisky Adherence Scale (*r_pmcc_* = 0.174, *p* = 0.002) in outpatients with stable schizophrenia (7) and the PDRQ-9 score was found to be significantly predicting adherence to medical cannabis in patients with chronic pain visiting a clinic for medical cannabis prescription (*p* < 0.01) (33).

### Construct validity – comparison with other questionnaires

Several studies compared the PDRQ-9 score with another questionnaire for the PDR, such as the Difficult Doctor-Patient Relationship Questionnaire (DDPRQ-10), a scale that assesses the doctor's view of the PDR, with which the PDRQ-9 score was correlated negatively (*r* = −.22, *p* = .003) (6); the Trust in physician scale (*r* = 0.413, *p* < 0.001), with which it correlates positively (7); and satisfaction with pain treatment (*r* = .51) (11). These correlations suggest moderate, acceptable construct validity.

### Reliability

All studies found the questionnaire reliable, with Cronbach's alpha ranging from 0.62 to 0.97 (mean 0.91). Test-retest reliability ranged from 0.61 over two months to 0.74 after seven days.

### Factor structure

Table 1 provides factor structure findings of 14 studies, including 11 of the validation studies (1, 7–17, 29, 135).

All studies confirmed the one-factor model of the PDRQ-9 except one study, which found a two-factor solution for the Chinese version. The first factor reflects empathy and trust experienced by the patient during treatment, and the second factor represents the patients' appraisal of the process and results of their treatment (14).

### Field studies with total scores

Table 3 provides the total scores of the PDRQ-9 and other findings from 62 publications describing field studies conducted with psychometrically tested versions of the PDRQ-9. They involved 25,865 patients from 24 countries: Austria, Bangladesh, Brazil, Canada, China, Germany, Ethiopia, Iran, Israel, Italy, Malaysia, the Maldives, Mexico, Nigeria, Oman, Romania, Saudi-Arabia, Spain, Taiwan, Thailand, the Netherlands, Turkey, the United Kingdom and the USA. All studies conducted are shown; in case studies do not show total scores, weighted Mean and SDs are reported.

Most studies were based on convenience samples in clinical settings and one Chinese study reporting on a convenience sample derived from an online survey conducted via internet and social media (75). In addition, some studies were performed in representative samples. Four publications covered one representative general population sample in Germany (11, 61–63), one study used a systematic random sampling method resulting in 372 surgical patients in Ethiopia (30) and one Brazilian study was nested within a national cross-sectional study conducted to assess the quality of care provided by the Brazilian Primary Health Care (PHC) system, with units being selected in each municipality using a systematic sampling methodology (71).

### Language versions and countries where the studies were conducted

Six studies in 957 patients were conducted with the Dutch version (1, 29, 41–44). Five studies of those reported total scores in 785 patients. Thirteen studies in 1,920 patients were conducted with the English version: eight in the USA (6, 22, 46–49, 52, 53); one in the UK; (45) in Canada, the USA and the UK (32); in Nigeria (7), Saudi Arabia (50), and India (51). Ten studies of those reported total scores in 1,490 patients. Seven studies were conducted in 3,677 patients with the Spanish version; six studies used the version psychometrically tested by Martín-Fernández et al. (2,010) (8) and were conducted in Spain (8, 9, 37) and in Mexico (23, 54, 55). One study (56) used the Mexican Spanish translation (10). Five studies of those reported total scores in 2,788 patients. Three Italian studies in 545 patients used the Italian version (57–59). The Romanian version was used in a study conducted in Romania in 55 children and adolescents (60). Eight studies encompassing 2,542 patients used the German version. Four of them reported on the same German representative general population sample rating their primary care doctor (11, 61–63); three more studies were conducted in other German samples (64–66) and one study was conducted in Austria (67). Five studies of those reported total scores in 2,542 patients. One study on the Hebrew version was performed in 93 patients in Israel (33). Two studies with the Arabic version were performed in 1,053 patients in Saudi Arabia (68) and Oman (69). The Persian version was conducted in three studies in 683 patients in Iran (16, 76, 77). Two studies reporting on the Bangla version in 264 patients were performed in Bangladesh (32, 70). The Thai version was used in four studies conducted in 1,205 patients in Thailand (14, 24, 62, 72). Two studies of those reported total scores in 423 patients. The Malay version was used in one study conducted in 906 patients in Malaysia (13). And the Maldivian version was used in the Maldives in 172 patients (9). The Chinese version was used in seven studies in 4,555 patients: five conducted in China (14, 15, 25, 26, 75), one in the Inner Mongolia Autonomous Region of People's Republic of China (74) and one in Taiwan (73). The Afaan Oroma version was used in one study in 372 patients in Ethiopia (30). Two studies on the Portuguese version were conducted in 6,921 patients in Brazil (15, 71).

**Figure 2:**
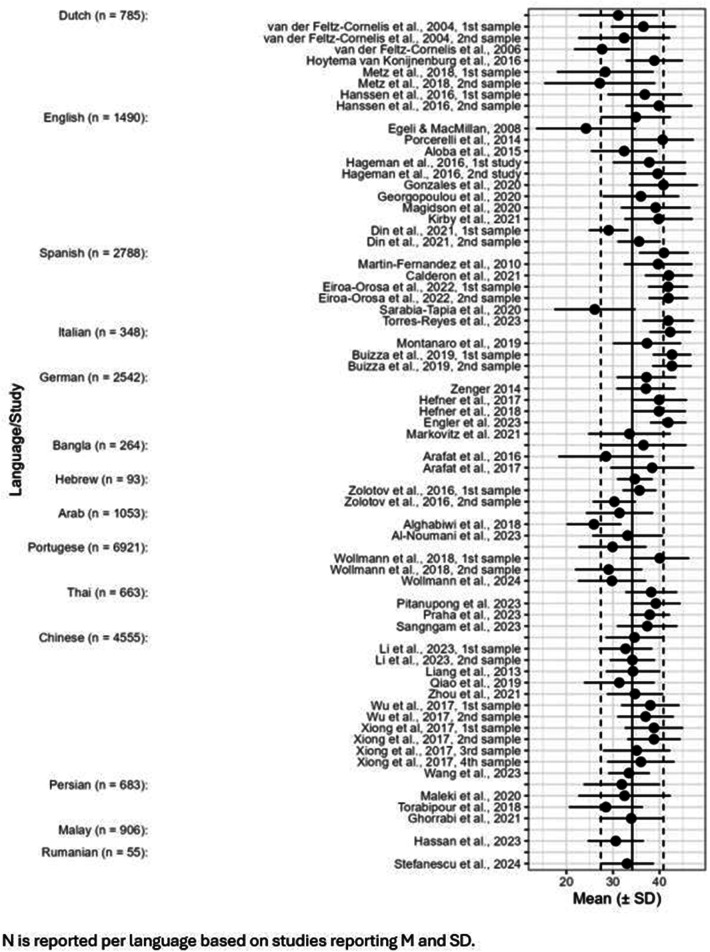
Forest plot with weighted means and pooled DSs for the total score per study grouped per language/country.

For 23,343 patients from 50 publications describing 63 samples in 14 languages, the overall weighted mean for the total score on the PDRQ-9 was *M_weighted_* = 34.21, *SD__pooled_* = 6.70. A forest plot based on total scores involving those 63 samples is shown in Figure 2.

As can be seen in Figure 2, the vertical line in the middle is the weighted total mean of all studies. The N per language is reported based on studies reporting an M and SD. The vertical striped lines on the left and on the right represent the SD limits of the weighted total means. The plot is arranged per country and the first dot per country represents the total for that country. As can be seen in the figure, the weighted total mean for the Dutch, English, Hebrew and Chinese versions correspond with the weighted total mean of all studies. The Spanish, Italian, Bangla and Thai version have a higher weighted total mean score. The other countries have a lower weighted total mean score.

### Ceiling effect

Many studies found that the PDRQ-9 presented discrimination problems in the upper part of the scale (8, 9, 11, 23, 38, 56, 61–65, 72). They found a clear left-skewed distribution, indicating that most values were concentrated on the right of the mean. This reveals a strong tendency to use the highest response options of the PDRQ-9 items and rate one's doctor positively. This led to skewed item scores and a skewed instrument's sum score. The latter is shown in Supplementary Figure A1 in the Supplementary Materials with a histogram for PDRQ-9 total scores of the German sample (11). The left side of Supplementary Figure A1 shows the distribution of PDRQ-9 total scores. Based on Rankit normalisation we converted the PDRQ-9 total score to normalised *T*-scores, whose distribution is shown on the right side in Supplementary Figure A1.

**Figure 3:**
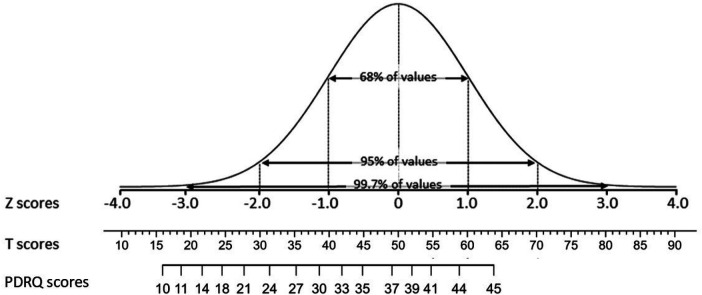
Normal distribution with *Z*-scores, *T*-scores, and raw scores on the PDRQ-9 total scale.

The relation between raw scores on the PDRQ-9 and *T*-scores under the normal distribution is shown in Figure 3.

The resulting PDRQ-9T-score metric is especially apt at distinguishing severity levels among the mildly to severely challenged PDRs in the lower realm.

### Item scores

19 Studies provided mean item scores of 14,486 patients (1, 7–9, 11, 12, 15, 16, 49–51, 54, 68, 70, 71, 73, 75–77) and 6 studies provided frequencies of response item options (1, 11, 50, 51, 70, 71). An overview of weighted mean item scores and SD__pooled_ is shown in Table 4.

The table shows the skewed representation of the PDRQ-9 item scores. Higher response options (“Appropriate”, “Mostly”, and “Totally”) were more frequently chosen than lower response options (“Not at all” and “Somewhat”). Furthermore, Table 4 shows data on the frequency of endorsed response options for a total of 14,697 patients as provided by twenty-one studies. (1, 7–9, 11, 12, 15, 16, 41, 49–51, 54, 68, 70–73, 75–77).

The weighted mean scores and pooled SD's for items are shown in Supplementary Table A1.

### Cut-off scores

Several studies suggested single cut-off values for a good PDR: 31 in Brazil (13, 71); >33.57 in Mexico (23); >34.5 in Ethiopia (30); and ≥39.69 in Spain (8), which is a much higher value than the others.

Other studies suggested cut-off values for distinct levels of severity, defining good and low PDR scores as 29 and ≤22 in Mexico (55); as ≥43.4 and ≤30.4 in the USA (53); as ≥36; 18–35 (moderate PDR); and ≤17 in Saudi Arabia (50) and Thailand (38, 72); as >36, 22–35 (moderate PDR) and ≤21 in Iran (77); ≥33, 21–32 (moderate) and ≤20 in the Maldives (35).

One study, conducted in Iran, suggested four levels with cut-off values: ≥38 for excellent “communication skill”, 29–38 for good, 19–28 for moderate, and 9–18 for weak “communication skill” (76).

Based on these reported cut-off scores and given the ceiling effect in the data of the studies, resulting in skewed sum scores on the instrument, distinguishing levels above average and providing a cut-off score is hard to do. Therefore, we provided norms that are especially useful for distinguishing levels below average or challenged PDRs. To this effect, we transformed raw scores from the German general population sample (11) to normalised *T*-scores and present these for all possible raw scores in the supplementary materials (Supplementary Table A2). This is a crosswalk table to convert raw PDRQ-9 scores to percentile rank order scores, normalised *T*-scores, and various levels of a challenged PDR. These T scores can also be established with a conversion formula (T = 0.61 + 1.885*RS-0.03865*RS^2^ + 0.0006151*RS^3^, a 3rd-grade polynomial), have a mean of M = 50.0 and SD = 10.0, and are equidistant. Scores below 30 (raw score <23) indicate an exceptionally challenged relation, reported by <2% of the general population. The Supplementary Table A2 presents a crosswalk table for PDRQ-9 raw scores to *T*-scores and Percentile Rank order (PR) scores.

Based on these normalised *T*-scores we recommend the following cut-off values as shown in Table 5: A cut-off value of T ≤ 44 for a challenged PDR, 45 ≤ T ≤ 56 for an average PDR, and T > 56 for a good PDR.

The studies give a range of cut-off scores for severely challenged PDR ranging from 17.00 to 39.69. The relation between raw scores, percentile ranks, and *T*-scores is also depicted in Supplementary Figure A2. *T*-scores are on the *x*-axis, percentile rank-order scores are on the *Y*-axis, and raw scores are shown in the figure with a white background on the sigmoid curve. Scores in the green area (RS ≥ 35) represent average to good PDRs, and scores in the red area (RS ≤ 30) indicate challenged PDRs. The figure shows how the raw scores in the white squares correspond to *T*-scores on the *X*-axis and percentile scores on the *Y*-axis. The red area indicates challenged PDR scores.

The scoring instructions and formula for conversion of raw scores to standardised, normalised scores is included in the Dutch and English version of the PDRQ-9 in the Supplementary Materials.

Several studies explored challenged PDRs in the following groups as indicated by lower total scores.

### Medically unexplained symptoms

In terms of medically unexplained symptoms, one study in primary care found that MUS patients reported significantly higher levels of loneliness and a lower quality of the PDR than older patients with explained medical symptoms (*p* = 0.002) (46). When taking into account comorbid depressive and anxiety disorders, only the association between MUS and the quality of the PDR remained statistically significant. The effect size of the association was small, with a beta of 0.179–0.191. *p* = 0.005–0.009. A Chinese study found that patients with mixed somatic and psychological distress had the worst PDR (*M* = 36.0; *SD* = 7.3). In addition, compared with patients with somatic distress alone, those with psychological distress alone had a worse PDR (73). Lower than average scores were found in fibromyalgia patients (32), and in primary care patients with persistent unexplained symptoms in primary care with high rates of comorbid anxiety and depressive disorders (41).

### Mental health conditions

Regarding mental health conditions, average PDRQ-9 scores were lower in a study in the Netherlands in the specialised mental health setting (43). The PDRQ-9 was found to be negatively correlated with the PHQ-9 (*r* = −0.196 to −0.309) and there were significant differences in PDRQ-9 ratings between general hospital in-patients with or without depressive symptoms (*p* = 0.019) in a Chinese study. This correlation was independent of age (14). Modest positive correlations were found of the PDRQ-9 with functional (*p* < .001) and global quality-of-life (*p* < .001), and negative correlations of the PDRQ-9 with psychological distress (*p* < .001) in oncology patients receiving treatment in specialist general hospital centres. Also, low PDRQ-9 scores for the PDR with the oncologist were associated with more anxiety (*p* = .006) and depression (*p* = .004) (9). In a general population sample, adjusted for age, the mean PDRQ-9 score of participants with a potential depressive disorder was *M* = 3.66 (*SD* = .86), and that of participants without a potential depressive disorder was *M* = 4.12 (*SD* = .66) (F = 65.8, p.001), a difference with a small effect size (Partial eta2 = .05). Experiencing somatic and depressive symptoms emerged as the most relevant patient-related predictors of the quality of the PDR with the family physician (11).

### Treatment factors

In terms of factors influencing treatment, in a Dutch study, the PDRQ-9 rating of the quality of the PDR was the only variable among patients' characteristics which was significantly and positively correlated (Pearson rho = −0.49; *P* < 0.01) with decisional conflict (42). Two other studies corroborated that the only factor associated with decisional conflict was the PDR (46, 47). A good understanding of the patients' illness was linked to a better relationship with their doctor and greater participation in shared decision making which was associated with increased trust and adherence to medication in another study (45). A study in psychiatric patients of Hospitals in Istanbul found a correlation between irregular attendance of the mental health services, and lower PDR (135). A Spanish study found a positive association (*r* = 0.38); *p* < 0.001) between consultation time and PDRQ-9 score: for every minute increase in consultation time, the PDRQ-9 score increased in 0.04 units (*p* = 0.001, CI95%: 0.016–0.063) (56).

PDRQ-9 scores were positively correlated with medication adherence rates for 3 months of treatment (*r* = 0.52; *P* = 0.006). Subjects in a US study who perceived a weaker PDR (less than or equal to 36, *n* = 10) were less adherent over 3 months, with an average adherence rate of 45%, compared with subjects who perceived a stronger PDR (greater than or equal to 37, *n* = 17), with an average adherence rate of 70% (*P* = 0.03). Adherence did not vary by age or gender (*P* = 0.59 and 0.51, respectively) (53). Several other studies found a positive correlation between PDRQ-9 score and medication adherence (35, 69). Regarding online treatments, internet hospital users who received online consultations rather than face to face gave significantly lower scores than regular hospital users (*P* = .01) (15).

### Gender

A Spanish study found that men had significantly higher PDRQ-9 scores than women (9). In Saudi Arabia, a female sample with chronic disease had an overall level of the PDR that was less than moderate. The PDR had a significant influence on the patients' self**-**efficacy in self**-**managing their chronic diseases (*p* = 0.047) (68). A study involving trans and non-binary patients found that non-binary participants showed a lower score (*M* = 30.79; SD = 7.46) than trans women (*M* = 34.78; SD = 9.17) and trans men (*M* = 36.10; SD = 9.45), a significant difference in means (F = 3.52, df = 2, *p* = 0.034) (67). The gender of the physicians was reported in none of these studies.

### Age

The number of studies exploring the role of age in the PDR was limited. One study in primary care found no significant correlation of the PDR with age, health, or psychological distress (6); another study found higher age (OR 1.03, 95% CI: 1.02–1.05) was associated with above average PDR (8). However, given the OR of 1.03, this effect can be considered to be clinically irrelevant.

## Discussion

Over the last 20 years, 66 studies used the PDRQ-9. Twenty-five studies translated and psychometrically tested the PDRQ-9 in 15 languages and 10,321 patients in healthcare settings ranging from primary care to outpatient and inpatient general hospital care, including specialty mental healthcare, and patient populations, providing psychometric information.

In general, the main psychometric aspects to be evaluated, as listed by the COSMIN guideline are: Content and Construct Validity, Structural Validity, Cross-cultural Validity, Responsivity, Test-retest Reliability, Internal Consistency Reliability and standard error of measurement (136). Content and Construct Validity of the PDRQ-9 is supported by the original Dutch study and the research with the English translation. Structural Validity, that is, factorial invariance between subgroups, was supported by five studies (9, 11, 14, 26, 31). Criterion validity, e.g., whether patients with lower PDRQ-9 scores have lower compliance with treatment, or less adherence to medication, is supported by sixteen studies (7, 17, 33, 35, 38, 45, 53, 55, 64, 69, 74, 96, 102, 103, 129, 135). Cross-cultural validity was the prime research question in all studies investigating translation of the PDRQ and was mostly supported by replicating the positive psychometric properties of the Dutch original. In all studies that explored the factor structure but one, the PDRQ-9 is reported to consist of one factor, and the internal consistency reliability is excellent.

In view of the requirements suggested in the COSMIN guideline (136), current gaps in the literature regarding psychometric research of the PDRQ are test-retest reliability that is only reported by five studies (1, 6, 7, 9, 14); and responsiveness, indicating if the score changes after an intervention aimed at improving the relationship, which is reported by one study (43). These gaps could be welcome topics for future research.

Although the PDRQ-9 predates the distinction between Patient-Reported Outcome Measures (PROMs) and Patient-Reported Experience Measures (PREMs), it is best considered a PREM, as it was specifically developed to assess the PDR as experienced and reported by the patient, which is a key aspect of the patient experience. It was conceptualised as a reflective measurement model in which all items are a manifestation of the same underlying construct, namely the PDR (136).

### Cross cultural validity of the different language versions

Based on Tables 1–3, the following can be resumed regarding cross-cultural validity of the various language versions of the PDRQ-9.

The original Dutch language version's validity was supported by psychometric analyses, its factor structure reported, and means and SDs reported in five out of six field studies. The English version was produced by forward – backward translation and consultation of native speakers; and psychometrically tested in USA, Canadian, UK and Nigerian studies. The one-factor structure of this English version was confirmed by confirmatory factor analysis performed in two of those studies, one in the USA and one in Nigeria (6, 7). This version was used in 11 field studies, 10 of which reported means and SDs. The original published English version can therefore be considered as extensively cross culturally psychometrically tested.

The German version was developed by forward backward translation and applied in a representative sample from the general population. Internal consistency and construct validity were explored and a CFA performed, which confirmed a one-factor structure. Eight field studies were executed, four of them in the general population sample and four in convenience samples, reporting means and SDs. Furthermore, the German version was at the basis of one of the Chinese language versions that was used in two studies, after an extensive translation and psychometric testing procedure (25, 26).

The Spanish version was developed in Spain, with three studies reporting reliability, two reporting validity and factor analysis. One Mexican Spanish version was also developed and investigated with CFA. Three field studies were performed in Spain and four in Mexico, reporting means and SDs.

The Thai version was developed by forward backward translation and CFA reported. Four field studies were done with three reporting means and SD. The Bengali version was developed by forward backward translation in Bangladesh. Factor analysis confirmed one component. Two field studies reported total means and SDs. The Persian version used forward backward translation, performed factor analysis and three field studies reported means and SDs. The Brazilian Portuguese version performed forward and backward translation and expert consultation, performed factor analysis and two field studies reporting means and SDs. The Arab version was made by forward-backward translation. The study was performed in Egypt and reported Cronbach's alpha but no factor analysis (31). Two field studies reported means and SDs, one in Saudi Arabia and one in Oman (68, 69).

Three Chinese versions have been used where it was unclear how these were translated (73–75). Li et al. reported translation method, Cronbach's alpha and factor structure indices aligning with a one-factor structure (15). Wang et al. developed a Chinese version by an extensive forward backward translation process and found support for a two factor structure with highly correlated factors (14). The authors note that their study is the only one reporting two factors and suggest that this might be related to a less individualistic culture in China compared to other countries, which may make Chinese people more apt to not only consider the actions of their doctor in the appraisal of the PDR, but also the contextual circumstances provided by the hospital. This study is the only one showing a cross-cultural difference in factor structure in all the studies performed with the PDRQ-9 so far, differentiating that Chinese version from the Li study. Seven field studies reported means and SDs: all in China, one in Taiwan and one in Mongolia, two of which were developed in collaboration with a German research group (25, 26). Those authors confirmed in writing that they followed an extensive translation process and that CFA confirmed a one-dimensional factor structure and high reliability, which was however only partly published. The remaining studies (73–75) used translations with unknown factor structure.

The following language versions may need further validation and field studies to be conclusive: The Turkish version was developed by expert involvement and CFA reported; another study also reported EFA. However, no field studies reporting means and SDs were done. The Ethiopian study developed an Afaan Oromo Version by forward backward translation and explored internal consistency, but did not report factor analysis or a field study with total means and SDs (30). The Hebrew version used forward and backward translation and reported construct validity and reliability, and one field study with means and SDs. The Malay and the Maldivian versions do not provide results regarding the factor structure. Three studies report on means and SDs of an Italian version of which development, validity and factor structure is not described; one Romanian study also describes means and SDs but no other information regarding development and validity of the Romanian version was provided.

In summary, this shows extensive cross-cultural validity for the factor structure in 9 language versions, and 7 language versions that are well underway and for which more work may be needed. In that respect, it is relevant to mention that an officially translated Italian version is currently developed in a study funded by the European Union. The picture emanating from this shows good cross cultural validity. In total, studies on the PDRQ-9 encompass four out of five continents with overwhelmingly similar structure and results.

Based on our findings, it seems that one-dimensionality of the PDRQ-9 is cross-culturally supported. One Chinese study does not support that, however, the correlation between the two factors that they find is very high (14). The other Chinese studies either do report a one-factor structure (15, 25, 26), or do not report a factor structure at all (73–75).

Another aspect that warrants attention is the variability in mean scores across studies. These differences may be attributable to variations in study populations (e.g., general population vs. clinical samples), characteristics of health care systems, translation-related issues, and broader cultural factors. Given the present data, it is difficult to disentangle the relative contributions of these factors. Therefore, norming based on representative samples from the general population is recommended.

Total scores of the PDRQ-9 are provided in 50 publications describing studies conducted in 63 samples, 23,343 patients and 24 countries, using psychometrically tested versions of the PDRQ-9. The overall weighted mean for the total score on the PDRQ-9 was *Mweighted* = 34.21, *SD_pooled* = 6.70. The weighted mean average varies between countries, with scores higher than average for studies conducted in the UK and USA, the Netherlands, Germany, Spain, the Northern part of Italy, and Thailand. This may reflect good health services in those regions compared to means in Hebrew, Chinese, Persian, and Romanian studies, which corresponded more to the weighted mean of the entire set of studies. Studies with the Arab, Romanian, Brazilian (Portuguese), Mexican and Malay versions yielded lower weighted mean scores. There are several possible explanations for this.

First, although most studies were performed with thoroughly translated and psychometrically tested language versions, the weighted total means scores of the Arab version seem to be afflicted by one study that was performed before the rigorously developed Arab version was published. The mean score reported in that study was substantially lower (68). This may be related to the quality of that translation, or with the population of patients with chronic medical conditions. The same may apply to the Romanian study, as it has an unclear translation process; however, given that the Romanian version clearly shows improvement in PDRQ-9 score after an intervention actually underscores the validity of this language version (60).

Second, it could reflect variations in health care service. For example, the M and SDs in the Brazilian sample are much lower than in other studies using the PDRQ-9. Methodological factors could play a role given that in this rural sample, comprising many illiterate patients from impoverished neighbourhoods, the PDRQ-9 was administered by interview, which might make people lean more toward average answers rather than at the high end of the scale, as in other studies that used the PDRQ-9 as a self-report measure. It is good to know that the interview method of administration allows for the valid appraisal of the PDR in deprived areas, but this difference in assessment may have influenced the completion of the PDR. Furthermore, relevant cultural and socio-economic factors could be that this sample contained many people who were illiterate and from impoverished neighbourhoods. The potential influence of such aspects of the primary healthcare system under study in Brazil on the PDR is captured effectively by the PDRQ-9.

However, it could also indicate PDR differences among patient populations. The findings support the known group validity of the PDRQ-9. Average scores tend to be lower in patients with chronic medical conditions (68) including Parkinsons disease (54). For example, the lower scores for the Arab version and the Mexican version might be partially explained by that.

Medically unexplained symptoms (MUS) and comorbid depressive and anxiety disorders, including fibromyalgia patients, are also found to be associated with lower scores (25, 32, 41, 44).

Dissatisfaction with the PDR in these patient groups is a long-standing issue, with many studies initially reporting on this from the perspective of doctors coining such patients as “worried well”, “difficult”, or “heartsink” patients (137–139). Most interventions in these patients would follow a cognitive behavioural treatment approach (140) which would be effective in patients willing and able to follow this treatment. However, studies show that overall the effect size of this approach tends to be small (141) and CBT provision as primary treatment was met over the years with growing discontent of patient groups, with a more recent development being studies with patient-input leading to redefining MUS as medically not yet explained symptoms (MNYES) (142) and emphasizing the need to study aetiology of syndromes, such as for example in chronic fatigue syndrome (143). Given this, the finding that the PDRQ-9 total score is lower than average in several studies in patients with MUS underscores the validity of this questionnaire and the relevance to explore the patient perspective of the PDR in the context of research. This may be supported by the finding of the lower score in the Malay study conducted in people visiting complementary medicine providers, possibly because of dissatisfaction with their regular healthcare providers (34).

Regarding patients with mental health issues, there seems to be a clear effect on the PDR in case of depression or anxiety, especially for patients who receive treatment from medical specialists in the general hospital, for example oncology patients (9); in specialty mental health patients with schizophrenia in Nigeria (7), and in patients with somatoform disorders in the Netherlands (41).

### Implications of the study

Our findings show that PDR is considered a relevant factor for patient well-being, compliance with treatment, and healthcare delivery in various countries, healthcare settings, and medical specialisations. The PDRQ-9 is especially apt in identifying a challenged PDR, which is the most relevant domain, making it a valuable tool for improving this critical aspect of care. We recommend reporting total scores rather than item scores and applying the normalised *T*-scores in future studies of the PDRQ-9 and in clinical practice. It could be beneficial to use the PDRQ-9 to screen for potential issues in the PDR in patient groups struggling with unexplained symptoms or mental health issues. This is more relevant as the PDRQ-9 score is associated with other factors influencing treatment, such as decisional conflict, medication adherence, and patients' self-efficacy in managing their chronic disease. The PDRQ-9 can also discern the quality of the PDR in online treatment settings (15) which indicates potential usefulness in evaluating the PDR as experienced by patients in e-Health interventions with the PDRQ-9.

Gender matters for how the PDRQ-9 is completed: Men rate their PDR significantly higher than women. In addition, non-binary participants have lower scores than trans women and trans men, who score average mean levels. However, so far, studies exploring gender have not taken the gender of doctors into account, which we recommend for future studies. Research should also explore which other doctor characteristics are associated with the PDRQ-9 score.

We presented a standardised scoring approach based on a large representative general population sample. This standardisation produces a normalised score that makes the raw scores that are cluttered due to the ceiling effect interpretable as it normalises the scale to equidistant scores. We present this as an example of how raw scores can be normalised. We suggest to apply this standardisation normalisation method of total scores, and the reported cut-scores, when using the PDRQ-9.

Furthermore, we recommend to perform general population studies to confirm norm scores in countries with mean scores diverting from the mean as displayed in Figure 2. New language versions of the PDRQ-9 should preferably be developed using the forward backwards translation method, and report on psychometric aspects including the factor structure.

### Limitations of the study

Limitations of this study are that there was only one representative sample of the general population. Most studies used convenience samples, although they reported on relevant clinical samples. Few studies have reported on gender differences among patients or potential relevant physician factors affecting PDR, such as educational levels, years of experience, gender, and age.

Although the single-factor structure is supported in most primary studies included in this systematic review, a direct cross-cultural comparison or Differential Item Functioning (DIF) analysis of configural, metric, and scalar factorial Invariance among translated versions for different nations or cultures is lacking. Only one of the primary studies assessed configural, metric and scalar DIF within one dataset/country (14), but none have done this across nations or cultures. We recommend inclusion of the PDQR-9 in future cross-country surveys such as the People Living with Chronic Conditions study (PaRIS) (144, 145), the Commonwealth Fund's International Health Policy survey (146), or other international initiatives, as this would secure representative data in many countries, and provide a basis for further research into normative data and generalizable results.

Another limitation concerned the utilization of the Risk of Bias tool. Assessing cross-sectional studies involves unique Risk of Bias considerations, and a recent review found that none of the currently available tools comprehensively address all potential biases pertinent to cross-sectional studies (27). Following the recommendations from that review, we developed a tool specifically for this systematic review. Biases that we should address were representativeness of the sampling frame, non-sampling error from nonresponse or coverage, and whether the outcome, disease or characteristic under study was measured validly and reliably, which in this case was operationalised by the level of validation of the respective translated versions of the PDRQ-9 in the selected studies. This allowed us to establish that Risk of Bias was low in most studies. However, risk of bias was moderate in studies with the Italian, Thai and some Arab versions, which limits the generalisability of the results for those language versions. Nevertheless, all studies show similar results.

Furthermore, there is a lack of uniformity in how the PDRQ-9 is scored: most researchers calculate the recommended sum score, but some calculate the mean score of the nine items. We recommend reporting the sum score in the future to avoid this inconsistency. Finally, the PDRQ-9 has a substantial ceiling effect, translating into a diminished capacity to discriminate among patients who score high on the PDRQ-9. Cut-off scores, therefore, can best be made for lower scores reflecting challenged PDRs and based on normalised *T*-scores. We recommend applying the normalised *T*-scores in clinical use and future studies of the PDRQ-9.

### Strengths of the study

The strengths of this study are the large number of studies, the large total sample, and the diversity of the samples regarding cultural background, with four out of five continents of the world represented. The proposed normalisation of scores is innovative, and the use of normalised sum scores enhances the interpretability of PDRQ-9 sum scores even in case of ceiling effects.

## Conclusion

The PDRQ-9 is a brief tool that effectively assesses the quality of the doctor-patient relationship from the perspective of the patient. Its factor structure has been reported across cultures, it is easy to administer, and shows strong reliability. Its scores are linked to better treatment adherence and outcomes in a variety of medical settings and patient populations. It is used worldwide and clearly fulfils a need. Studies were performed in primary care and various specialist general healthcare and mental healthcare settings in up to 24 countries. Construct validity and criterion validity are well supported, with correlations between the PDRQ-9 score, a good understanding of the patient's illness, greater participation in shared decision-making, and treatment adherence. Ceiling effect was reported. However, the PDRQ-9 provides a good measure of the PDR when total scores are transformed to a metric with a normal distribution, yielding helpful information, especially when the PDR is challenged. Transformed, normalised total scores should be reported preferably over mean scores given the provided cut-off score levels and for consistency in the international literature. Given the importance of the PDR in health services, the PDRQ-9 is a highly relevant measure. Research exploring doctor's factors playing a role in the PDR is recommended.

## Data Availability

The data analyzed in this study is subject to the following licenses/restrictions: The results are based on open access publications. One dataset was obtained from the authors for analysis and can be obtained by other researchers in the same way. Requests to access these datasets should be directed to Prof. Dr. med. Winfried Häuser Medizinisches Versorgungszentrum für Schmerzmedizin und seelische Gesundheit Saarbrücken - St. Johann Health Care Center Pain Medicine and Mental Health Saarbrücken - St. Johann Großherzog-Friedrich-Straße 44 D-66111 Saarbrücken Deutschland (Germany) winfriedhaeuser@googlemail.com, https://www.researchgate.net/profile/Winfried_Haeuser.
