# Supplementary material for: The patient-doctor relationship questionnaire (PDRQ-9). An overview of 20 years of research and a proposal for normalisation of scores. Systematic review
Source: Front Health Serv. 2026 Feb 19;6:1754286. doi: 10.3389/frhs.2026.1754286 (PMC12960598; doi:10.3389/frhs.2026.1754286)
Supplement: Supplementary file 1 [file Datasheet1.pdf]

Supplemental file

Supplemental Figure A1. Raw scores on the PDRQ9 scale related to the T-scores scale that has a normal distribution on the normal curve.

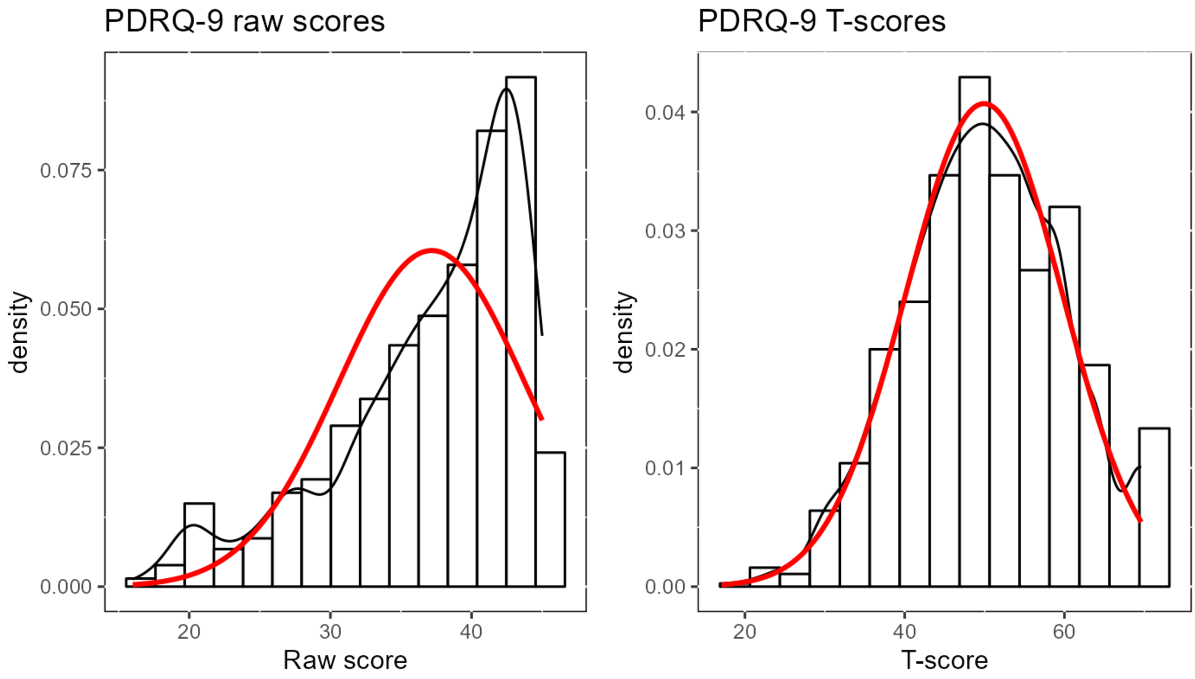

Supplemental Table A1

Study	N	M	SD
Dutch:	*785*	*31.15*	*8.45*
Van der Feltz-Cornelis et al, 2004, 1st sample[1]	110	36.54	6.93
Van der Feltz-Cornelis et al, 2004, 2nd sample[1]	55	32.40	9.81
Metz et al., 2018, 1st sample[50]	94	28.35	10.35
Metz et al., 2018, 2nd sample[50]	106	27.18	11.88
Hanssen et al., 2016, 1st sample[93]	107	36.84	7.98
Hanssen et al., 2016, 2nd sample[93]	150	39.86	7.14
Hoytema van Konijnenburg,et al. 2006[85]	82	38.88	6.12
English:	*1490*	*34.91*	*7.52*
Egeli & MacMillan, 2008[86]	190	24.22	10.66
Porcerelli et al., 2014[67]	180	40.67	6.64
Aloba et al., 2015[82]	309	32.40	7.09
Hageman et al., 2016,1st study[94]	84	37.80	7.83
Hageman et al., 2016, 2nd study[95]	84	39.60	6.03
Gonzales et al., 2020[8]	102	40.83	7.35
Georgopoulou et al., 2020[100]	98	36.00	8.19
Magidson et al., 2013[97]	77	39.16	7.43
Kirby et al., 2021[96]	218	39.80	7.30
Din et al., 2021, 1st sample[102]	73	29.08	4.21
Din et al., 2021, 2nd sample[102]	75	35.61	4.61
Spanish:	*2788*	*40.94*	*5.28*
Martin-Fernandez et al., 2010[73]	451	39.69	7.31
Calderon et al., 2021[75]	560	42.00	5.10
Eiroa-Orosa et al., 2023, 1st sample[69]	1002	41.84	4.25
Eiroa-Orosa et al., 2023, 2nd sample[69]	560	41.91	4.21
Sarabia-Tapia et al., 2020[103]	100	26.10	8.7
Torres-Reyes et al., 2023	115	41.87	5.46
Italian:	*545*	*42.29*	*4.43*
Montanaro et al., 2019[108]	24	37.30	7.30
Buizza et al., 2019, 1st sample[107]	164	42.67	4.10
Buizza et al., 2019, 2nd sample[107]	160	42.64	4.20
Lauricella et al., 2021	197	42.29	4.43
German:	*2542*	*37.19*	*6.35*
Zenger 2014[11]	2275	37.08	6.30
Hefner et al., 2017[111]	58	39.92	5.97
Hefner et al., 2018[110]	64	39.87	5.76
Engler et al. 2023[112]	54	41.85	3.87
Markovitz et al. 2021[113]	91	33.55	8.83
Bangla:	*264*	*36.55*	*7.70*

Study	N	M	SD
Arafat et al., 2016[81]	50	28.50	10.20
Arafat et al., 2017[118]	214	38.43	7.00
**Hebrew:**	*93*	*34.71*	*3.79*
Zolotov et al., 2016, 1st sample[76]	76	35.70	3.60
Zolotov et al., 2016, 2nd sample[76]	17	30.30	4.60
**Arab:**	*1053*	*31.41*	*7.18*
Alghabiwi et al., 2018[114]	253	25.92	5.84
Al-Noumani et al., 2023[115]	800	33.14	7.55
**Brazilian (Portugese):**	*6921*	27.47	6.39
Wollmann et al., 2018, 1st sample[74]	133	40.05	6.30
Wollmann et al., 2018, 2nd sample[74]	628	29.07	7.20
Wollmann et al., 2024[12]	6160	30.00	8.91
**Thai:**	*423*	*38.71*	*4.95*
Pitanupong et al. 2023[83]	264	39.20	5.30
Praha et al., 2023[80]	159	37.90	4.30
**Chinese:**	*4555*	*34.63*	*6.19*
Li et al., 2023, 1st sample[78]	130	32.71	5.72
Li et al., 2023, 2nd sample[78]	375	34.17	4.85
Liang et al., 2013[122]	256	34.30	5.80
Qiao et al., 2019[121]	713	31.36	7.56
Zhou et al., 2021[87]	1903	34.74	6.00
Wu et al., 2015, 1st sample[23]	248	38.03	6.25
Wu et al. 2015, 2nd sample[23]	236	37.01	6.03
Xiong et al. 2017, 1st sample[120]	202	38.80	6.25
Xiong et al. 2017, 2nd sample[120]	89	38.80	5.70
Xiong et al., 2017, 3rd sample[120]	51	35.10	7.20
Xiong et al., 2017, 4th sample[120]	149	36.00	7.20
Wang et al., 2023[84]	203	33.45	4.44
**Persian:**	*683*	*31.90*	*8.17*
Maleki et al., 2020[72]	208	32.49	9.90
Torabipour et al., 2018[116]	200	28.50	7.91
Ghorrabi et al., 2021[117]	275	33.92	6.80
**Malay:**	*906*	*30.60*	*5.99*
Hassan et al., 2023[71]	906	30.60	5.99
**Rumanian:**	*55*	*33.02*	*5.74*
Stefanescu et al., 2024[109]	55	33.02	5.74
**Total N, weighted mean, pooled SD**	***23343***	***34.21***	***6.70***

NB: Numbers in italics are the summed N, weighted mean, and pooled SD

Supplemental Table A2 presents a crosswalk formula for PDRQ-9 raw scores to T-scores and Percentile scores

RS	PR	T-Rankit	Meaning	RS	PR	T-Rankit	Meaning
9	NA	NA	severe	28	10	37.2	moderate
10	0	16.7	severe	29	12	38.1	moderate
11	0	19.4	severe	30	14	39.1	moderate
12	NA	NA	severe	31	16	40.1	mild
13	0	20.8	severe	32	19	41.1	mild
14	0	22.4	severe	33	22	42.3	mild
15	NA	NA	severe	34	26	43.7	mild
16	0	23.6	severe	35	32	45.2	average
17	1	24.3	severe	36	40	47.4	average
18	1	25.2	severe	37	48	49.4	average
19	1	26.2	severe	38	53	50.8	average
20	1	27.0	severe	39	59	52.3	average
21	2	28.3	severe	40	64	53.6	average
22	2	29.3	severe	41	69	55.0	average
23	2	30.3	moderate	42	73	56.3	average
24	4	32.0	moderate	43	78	57.6	good
25	5	33.3	moderate	44	82	59.0	good
26	6	34.2	moderate	45	92	63.9	good
27	8	35.8	moderate				

NB: RS = Raw Score; PR = Percentile Rank Score, T-Rankit = Percentile-based T-score; NA means that these scores were not present in the dataset.

Supplemental Table A3 Risk of Bias

Study	Target group described<br>Patients and doctors with which they have a PDR	Sampling method	Valid assessment of PDRQ-9	Selective reporting	Risk of bias
Dutch					
Van der Feltz-Cornelis et al, 2004[1]<br>The Netherlands	Y Primary Care patients and Primary Care Physicians; Epilepsy clinic inpatients and neurologists	Convenience sample	Y Dutch validated version of van der Feltz-Cornelis[1]	N<br>Total score and item means (±SD) and frequencies of response options per item provided	Low
Van der Feltz-Cornelis et al, 2006[91]<br>The Netherlands	Y Primary Care patients with somatoform disorders and Primary Care Physicians	Convenience sample	Y Dutch validated version of van der Feltz-Cornelis[1]	N<br>Total score and item means (±SD) and frequencies of response options per item provided	Low
Du Long et al., 2016[92]<br>The Netherlands	Y general hospital outpatients with either knee or hip osteoarthritis and orthopaedic surgeons	Convenience sample	Y Dutch validated version van der Feltz-Cornelis[1]	Y no Total score mean (±SD) provided	Moderate
Hoytema van Konijnenburg et al., 2016[85]<br>The Netherlands	Y parents of children outpatients of a general hospital and paediatricians	Convenience sample	Y Dutch validated version of van der Feltz-Cornelis[1]	N Total score mean (±SD) provided	Low
Metz et al., 2018[50]<br>The Netherlands	Y specialty mental health outpatients and mental health care professionals	Convenience sample	Y Dutch validated version of van der Feltz-Cornelis[1]	N Total score mean (±SD) provided	Low
Hanssen et al., 2016[93]<br>The Netherlands	Y Primary care patients > 60 years and primary care physicians	Convenience sample	Y Dutch validated version of van der Feltz-Cornelis[1]	N Total score mean (±SD) provided	Low
English					

Study	Target group described<br>Patients and doctors with which they have a PDR	Sampling method	Valid assessment of PDRQ-9	Selective reporting	Risk of bias
Georgopoulou et al., 2020[100]<br>UK	Y General hospital outpatients with lupus nephritis and nephrologists	Convenience sample	Y English translation of validated version provided by van der Feltz-Cornelis[1]	N Total score mean (±SD) provided	Low
Porcerelli et al., 2014[67]<br>Detroit, USA	Y Primary care patients and primary care physicians	Convenience sample	Y English translation of validated version provided by van der Feltz-Cornelis[1] and psychometric properties provided by Porcerelli[67]	N Total score means (±SD) provided	Low
Aloba et al., 2015[82]<br>Nigeria	Y Specialty mental health outpatients with stable schizophrenia and psychiatrists	Convenience sample	Y English translation of validated version van der Feltz-Cornelis[1,] , psychometric properties reported by Aloba[82]	N Total score and item means (±SD) provided	Low
Hageman et al., 2016 April[94]<br>USA	Y Orthopaedic hospital outpatients and orthopedic surgeons	Convenience sample	Y English translation of validated version provided by van der Feltz-Cornelis[1]	N Total score means (±SD) provided	Low
Hageman et al., 2016 Oct[95]<br>USA	Y Orthopaedic hospital outpatients and orthopedic surgeons	Convenience sample	Y English translation of validated version provided by van der Feltz-Cornelis[1]	N Total score means (±SD) provided	Low
Gonzalez et al., 2020[8]<br>USA	Y Orthopaedic hospital outpatients and orthopedic surgeons	Convenience sample	Y English translation of validated version of van der Feltz-Cornelis[1] and psychometric properties provided by Gonzalez[8]	N Total score means (±SD) provided	Low
Magidson et al., 2013[97]<br>USA	Y outpatients of a substance use disorder centre with HIV and their therapists	Convenience sample	Y English translation of validated version provided by van der Feltz-Cornelis[1]	N Total score means (±SD) provided	Low

Study	Target group described<br>Patients and doctors with which they have a PDR	Sampling method	Valid assessment of PDRQ-9	Selective reporting	Risk of bias
Kirby et al., 2021[96]<br>USA	Y Parent-child pairs in primary care practice and primary care physicians	Convenience sample	Y English translation of validated version provided by van der Feltz-Cornelis[1], adapted for children and parents	N Total score means (±SD) provided	Low
Alomran et al. 2020[101]<br>Saudi Arabia (English version)	Y primary care patients and primary care physicians	Convenience sample	Y English translation of validated version provided by van der Feltz-Cornelis[1]	Y no M (±SD) provided, but item means (±SD) and frequencies of response options	Moderate
Din et al., 2021[102]<br>India	Y Surgical and physiotherapy hospital outpatients and orthopaedic surgeons and physiotherapists	Convenience sample	Y English translation of validated version provided by van der Feltz-Cornelis[1]	N Item means (±SD) and frequencies of response options per item provided	Low
Versluijs et al., 2021[98]<br>Texas, USA.	Y hospital surgery outpatient and surgeons	Convenience sample	Y English translation of validated version provided by van der Feltz-Cornelis[1]	Y no M (±SD) provided	Moderate
Perche et al., 2023[99]<br>USA	Y dermatology hospital outpatients and dermatologists	Convenience sample	Y English translation of validated version provided by van der Feltz-Cornelis[1]	Y no M (±SD) provided	Moderate
Egeli & MacMillan, 2008[86]<br>Online (USA, Canada, UK)	Y fibromyalgia patients and physicians	Convenience sample	Y English translation of validated version provided by van der Feltz-Cornelis[1]	N Total score means (±SD) provided	Low
Spanish					
Martín-Fernández et al., 2010[73]<br>Spain	Y Primary care patients and primary care physicians	Convenience sample	Y Spanish PDRQ-9 validated by Martín-Fernández[73]	N Total score means (±SD) provided	Low
Calderón et al., 2021[75]<br>Spain	Y oncology hospital outpatients and oncologists	Convenience sample	Y Spanish PDRQ-9 validated by Martín-Fernández[73]	N Total score means (±SD) provided	Low

Study	Target group described<br>Patients and doctors with which they have a PDR	Sampling method	Valid assessment of PDRQ-9	Selective reporting	Risk of bias
Eiroa-Orosa et al., 2023[69]<br>Spain	Y Primary care patients and primary care physicians	Convenience sample	Y Spanish PDRQ-9 validated by Martín-Fernández[73]	N Total score means (±SD) provided	Low
Sarabia-Tapia et al., 2020[103]<br>Mexico	Y Neurological hospital outpatients and neurologists	Convenience sample	Y Spanish PDRQ-9 validated by Martín-Fernández[73]	N Total score means (±SD) provided	Low
Pascual – Ramos et al. 2022[9]<br>Mexico	Y Rheumatological hospital outpatients and rheumatologists	Convenience sample	Y Spanish PDRQ-9 validated by Martín-Fernández[73]	N Total score means (±SD) provided	Low
Peña-Valenzuela et al., 2022[104]<br>Mexico	Y Primary care patients and primary care physicians	Convenience sample	Y Spanish PDRQ-9 validated by Martín-Fernández[73]	Y no M (±SD) provided	Moderate
Torres-Reyes et al., 2023[105]<br>Mexico	Y Primary care patients and primary care physicians	Convenience sample	Y Mexican Spanish translated and validated by Fernández Castillo[77]	Y Total score mean (±SD) provided	Moderate
Fernández Castillo et al., 2021[77]<br>Mexico	Y Patients with Diabetes Mellitus and their treating physician	Convenience sample	Y Mexican Spanish validated by Fernández Castillo[77]	N Total score mean (±SD) provided	Low
Italian					
Montanaro et al., 2019[108]<br>Italy	Y Neurology hospital outpatients and neurologists	Convenience sample	Unclear	N Total score means (±SD) provided	Moderate
Buizza et al., 2019.[107]<br>Italy	Y Oncology outpatients and oncologists	Convenience sample	Unclear	N Total score means (±SD) provided	Moderate
Lauricella et al., 2021[106]	Y Neurology hospital outpatients and neurologists	Convenience sample	Unclear	N Total score means (±SD) provided	Moderate
Romanian					
Stefanescu et al., 2024[109]<br>Romania	Y Children and adolescents with Type I diabetes and their treating physician	Convenience sample	Romanian translation of the English translation of validated version	N Total score means (±SD) provided	Low

Study	Target group described<br>Patients and doctors with which they have a PDR	Sampling method	Valid assessment of PDRQ-9	Selective reporting	Risk of bias
			provided by van der Feltz-Cornelis[1]		
German					
Dinkel et al., 2016 Maart[89]<br>Germany	Y People in the general population and their primary care physician	Representative sample of the German general population	Y German PDRQ-9 validated by Zenger[11] from the Dutch validated version van der Feltz-Cornelis[1]	N Total score mean (±SD) provided	Low
Dinkel et al., 2016 July[88]<br>Germany	Y People in the general population and their primary care physician	Representative sample of the German general population	Y German PDRQ-9 validated by Zenger[11] from the Dutch validated version van der Feltz-Cornelis[1]	N Total score mean (±SD) provided	Low
Schmalbach et al, 2024[90]<br>Germany	Y People in the general population and their primary care physician	Representative sample of the German general population	Y German PDRQ-9 validated by Zenger[11] from the Dutch validated version van der Feltz-Cornelis[1]	N Total score mean (±SD) provided	Low
Zenger et al., 2014[11]<br>Germany	Y People in the general population and their primary care physician	Representative sample of the German general population	Y German PDRQ-9 validated by Zenger[11] from the Dutch validated version van der Feltz-Cornelis[1]	N Total score mean (±SD) and frequencies of response options per item provided	Low
Hefner et al., 2017[111]<br>Germany	Y Oncology outpatients and oncologists	Convenience sample	Y German PDRQ-9 validated by Zenger[11] from the Dutch validated version van der Feltz-Cornelis[1]	N Total score mean (±SD) provided	Low
Hefner et al., 2018[110]<br>Germany	Y Oncology outpatients and oncologists	Convenience sample	Y German PDRQ-9 validated by Zenger[11] from Dutch validated	N Total score mean (±SD) provided	Low

Study	Target group described Patients and doctors with which they have a PDR	Sampling method	Valid assessment of PDRQ-9	Selective reporting	Risk of bias
			version van der Feltz-Cornelis[1]		
Engler et al. 2023[112] Germany	Y Primary care patients and primary care physicians	Convenience sample	Y German PDRQ-9 validated by Zenger[11] from the Dutch validated version van der Feltz-Cornelis[1]	N Total score mean (±SD) provided	Low
Markovic et al. 2021[113] Austria	Y Transgender clinic outpatients and plastic surgeons	Convenience sample	Y German PDRQ-9 validated by Zenger[11] from the Dutch validated version van der Feltz-Cornelis[1]	N Total score mean (±SD) provided	Low
Hebrew					
Zolotov et al., 2016[76] Israel	Y Chronic pain clinic outpatients and physicians	Convenience sample	Y Hebrew translation of the English version of van der Feltz-Cornelis[1] and adapted to reflect PDR in case of use of medical cannabis by Zolotov.[76]	N Total score mean (±SD) provided	Low
Turkish version					
Deniz et al., 2021[7] Turkey	Y Primary care patients and primary care physicians	Convenience sample	Y Turkish translation and validation of the English version of van der Feltz-Cornelis[1] by Deniz.[7]	N Total score mean (±SD) provided	Low
Bener et al., 2025 Turkey	Y	Convenience sample	Y Turkish PDRQ-9	N	Low
Arab					
Alghabiwi et al., 2018[114] Saudi Arabia	Y Female primary care patients and primary care physicians	Convenience sample	Unclear	Y no Total score M (±SD) provided	Moderate

Study	Target group described Patients and doctors with which they have a PDR	Sampling method	Valid assessment of PDRQ-9	Selective reporting	Risk of bias
Al-Noumani et al., 2023[115] Oman	Y general hospital outpatients and medical specialists	Convenience sample	Unclear "Arabic version" (no further information provided)	Y No total score mean (±SD) provided, but item means (±SD)	Moderate
Hegazy et al., 2021[68] Egypt	Y Primary care patients and primary care physicians	Convenience sample	Arabic translation and validation of the English version of van der Feltz-Cornelis[1]	Y No means/SDs for total score are items provided, but the aim of the study was to establish psychometric properties	Low
Bangla (Bengali)					
Arafat et al., 2016[81]	Y Specialty mental health outpatients and psychiatrists	Convenience sample	Y Bangla translation and validation of the English version of van der Feltz-Cornelis[1] by Arafat[81]	N Total score and item means (±SD) provided	Low
Arafat et al., 2017[118] Bangladesh	Y general hospital outpatients and medical specialists	Convenience sample	Y Bangla translationand validation of the English version of van der Feltz-Cornelis[1] by Arafat[81]	N Total score and item means (±SD) and frequencies of response options provided	Low
Brazilian (Portuguese)					
Wollmann et al., 2018[74]	Y Primary care patients and primary care physicians	Convenience sample	Y Brazilian (Portuguese) translation and validation of English version van der Feltz-Cornelis[1] by Wollmann[74]	N	Low
Wollmann et al., 2024[12]	Y Primary care patients and primary care physicians	Representative sample of the Brazilian primary care population	Y Brazilian (Portuguese) translation and validation of English version of van der Feltz-Cornelis[1] by Wollmann[74]	N Total score and item means (±SD) and frequencies of response options per item provided	Low
Thai					

Study	Target group described<br>Patients and doctors with which they have a PDR	Sampling method	Valid assessment of PDRQ-9	Selective reporting	Risk of bias
Pitanupong et al. 2023[83]<br>Thailand	Y University hospital outpatients with depression and psychiatrists	Convenience sample	Unclear<br>Thai translation of the English version of van der Feltz-Cornelis[1]	N	Moderate
Pitanupong et al. 2024[119]<br>Thailand	Y specialty mental health LBGTQ+ outpatients and their physicians	Convenience sample	Unclear	N	Moderate
Praha et al., 2023[80]<br>Thailand	Y Respiratory clinic outpatients and lung specialists	Convenience sample	Unclear	N	Moderate
Sangngam et al., 2023[10]<br>Thailand	Y Asthma patient and their treating physicians	Convenience sample	Unclear	Y No Mean (±SD) reported	Moderate
Chinese					
Li et al., 2023[78]	Y Hospital outpatients and their physicians	Convenience sample	Y Chinese translation and validation from English validated version van der Feltz-Cornelis[1] by Li[78]	N<br>Total score and item means (±SD) provided	Low
Liang et al. 2013[122]<br>Taiwan	Y Veterans and their health care providers	Convenience sample	Y Chinese translation validated from English validated version van der Feltz-Cornelis[1] by Liang[122]	N<br>Total score and item means (±SD) provided	Low
Qiao et al., 2019[121]<br>Inner Mongolia Autonomous Region of People's Republic of China	Y Hospital outpatients and their physicians	Convenience sample	Unclear	N Total score means (±SD) provided	Moderate
Zhou et al., 2021[87]	Y Hospital outpatients and their physicians	Convenience sample	Unclear	N<br>No total scores but item means (±SD) and frequencies of response options per item provided	Moderate
Wu et al., 2015[132]	Y Hospital outpatients and their physicians	Convenience sample	Unclear	N Total score means (±SD) provided	Moderate
Xiong et al., 2017[120]	Y Hospital outpatients and their treating physician	Convenience sample	Unclear	N Total score means (±SD) provided	Moderate

Study	Target group described Patients and doctors with which they have a PDR	Sampling method	Valid assessment of PDRQ-9	Selective reporting	Risk of bias
Wang et al., 2023[84]	Y Hospital outpatients and their treating physician	Convenience sample	Y New Chinese translation and validation of the English version of van der Feltz-Cornelis[1] by Wang[84]	N Total score means (±SD) provided	Low
Persian					
Maleki et al.,2020[72] Iran	Y Primary care patients and primary care physicians	Convenience sample	Y Persian translation and validation of the English version of van der Feltz-Cornelis[1] by Maleki[72]	N Total score and item means (±SD) provided	Low
Torabipour et al., 2018[116] Iran	Y Primary care patients and primary care physicians	Convenience sample	Unclear	N Total score and item means (±SD) provided	Low
Ghorrabi et al., 2021[117] Iran	Y Hospital outpatients and cardiologists, gynaecologists, ophthalmologists, Ear Nose Throat specialists and orthopaedic surgeons	Convenience sample	Unclear	N Total score and item means (±SD) and frequencies of response options per item provided	Moderate
Malay					
Johny et al., 2017[70] Malaysia	Y Patients attending traditional and complementary medicine physicians	Convenience sample	Malay translation and validation of the English version of van der Feltz-Cornelis[1] provided by Johny[70]	N	Low
Afaan Oromo					
Biyazin et al., 2022[79] Ethiopia	Y Hospital outpatients and surgeons	Representative sample pf surgical patients	Afaan Oromo translation validated from English	N	Low

Study	Target group described Patients and doctors with which they have a PDR	Sampling method	Valid assessment of PDRQ-9	Selective reporting	Risk of bias
			validated version van der Feltz-Cornelis[1] by Biyazin[79]		
Maldivian					
Hassan et al., 2023[71] Maldives	Y primary care patients and primary care physicians	Convenience sample	Maldivian translation validated from English validated version van der Feltz-Cornelis[1] by Hassan[71]	Y no M (±SD) provided	Moderate

Based on [130] Shannon E. Kelly, Stephen P.J. Brooks, Karima Benkhedda, Amanda J. MacFarlane, Linda S. Greene-Finestone, Becky Skidmore, Tammy J. Clifford, George A. Wells. A scoping review shows that no single existing risk of bias assessment tool considers all sources of bias for cross-sectional studies. Journal of Clinical Epidemiology Volume 172, 2024, 111408, ISSN 0895-4356, https://doi.org/10.1016/j.jclinepi.2024.111408.

Van der Feltz-Cornelis et al.[1] developed the questionnaire in Dutch and included an English translation in their first publication. This English version has been used extensively and its validity was established in Anglo-Saxon contexts. Most studies used this English version as source for translation, with the exception of the German studies which utilized the Dutch version as source.

Supplemental Figure A2 The relation between raw scores, percentile ranks, and T-scores

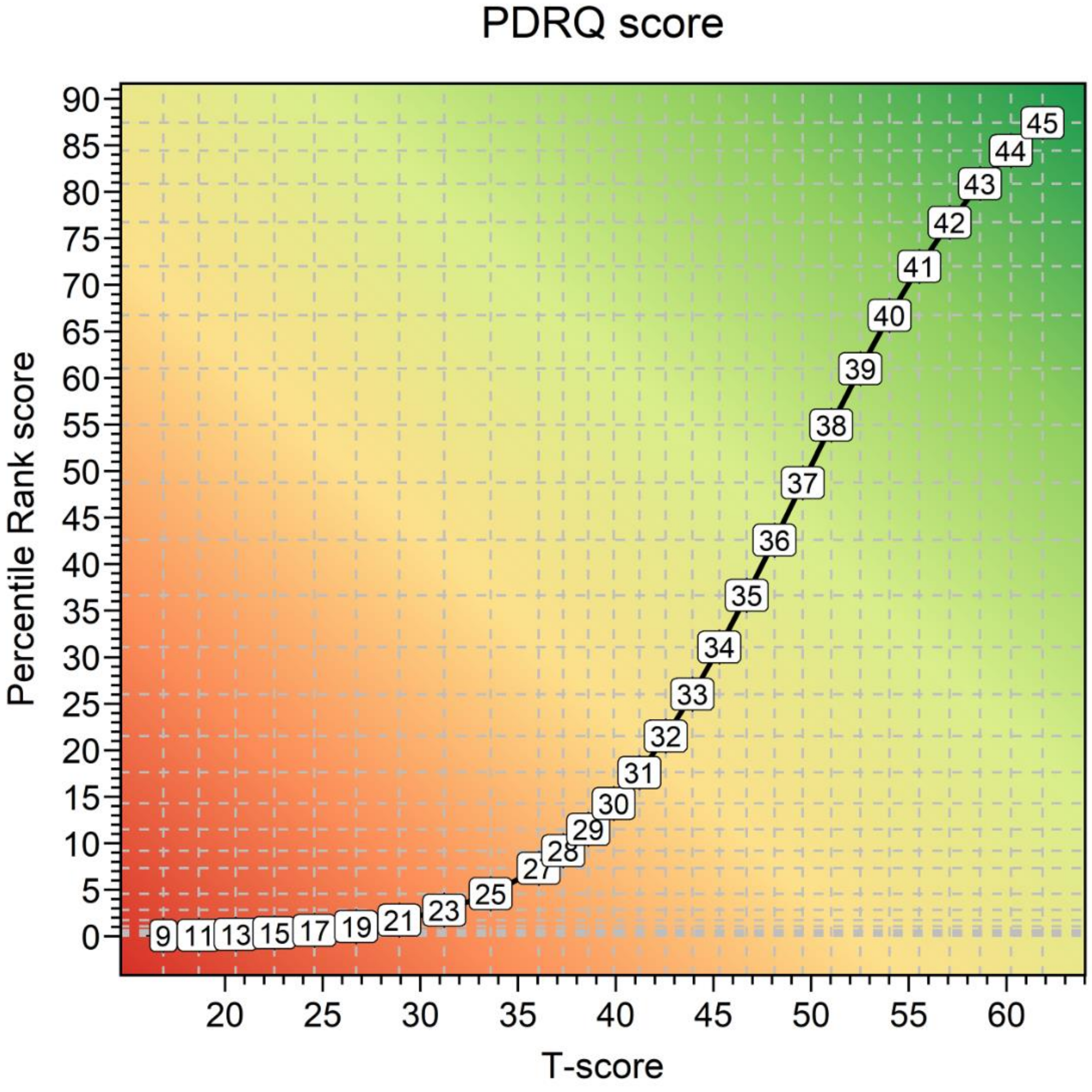

PDRQ-9 English version

Instruction:
You will read nine statements that a person can make about his/her doctor. Please choose the appropriateness of each statement for your doctor by marking one number per statement. The meaning of the numbers is as follows:

1 _ not at all appropriate
2 _ somewhat appropriate
3 _ appropriate
4 _ mostly appropriate
5 _ totally appropriate

1	My doctor helps me	1	2	3	4	5
2	My doctor has enough time for me	1	2	3	4	5
3	I trust my doctor	1	2	3	4	5
4	My doctor understands me	1	2	3	4	5
5	My doctor is dedicated to help me	1	2	3	4	5
6	My doctor and I agree on the nature of my medical symptoms	1	2	3	4	5
7	I can talk to my doctor	1	2	3	4	5
8	I feel content with my doctor's treatment	1	2	3	4	5
9	I find my doctor easily accessible	1	2	3	4	5
	Subtotal score per answering option (i.e. all 1s, all 2s etc.)					
	**Total score all items**					

Scoring
Item scoring
The questionnaire consists of 9 items assessing a single underlying construct: the patient's experience of the relation with their doctor. Each item is scored on a 5-point response scale, ranging from 1 to 5. All items are scored in the same direction; higher item scores indicate a more positive experience.

Handling of missing item responses
If no more than two items are missing (i.e., ≤ 22% of the items), the score may be calculated by replacing the missing item(s) with the mean of the completed items for that respondent.
If more than two items are missing, the total and mean scores should not be calculated and should be treated as missing.

Total score
A total score is obtained by summing the scores of all 9 items. The total score ranges from 9 to 45. This is a raw score (RS).

Interpretation of raw total scores and cut-off values
Higher total or mean scores indicate a better experience. For the raw score, we recommend cut-off values of RS < 35 for a challenged patient-doctor relationship (PDR), 35≤ RS ≤ 42 for an average PDR, and RS > 42 for a good PDR.

Standardisation of the raw total score

Standardized scores (T-scores) can be obtained after normalizing the raw scores with a polynomial transformation. T-scores have a mean of M = 50 and SD = 10. The function to transform a raw score (RS) to normalized T-scores is as follows:

$T_{normalized}$ = *6.1e-01+1.89****RS***-3.865e-02****RS***^2+6.151e-04****RS***^3.*

Example:<br>For an RS of **37** (the mean total score) this implies:<br>6.1e-01+1.89***37**-3.865e-02***37**^2+6.151e-04***37**^3 =<br>6.1e-01+1.89***37**-3.865e-02*1369+6.151e-04*50653 =<br>70.54 – 52.91 +31.15 = 48.78, which approximates T = 50.

This formula can be implemented in Microsoft Office Excel or other statistical/computational software, such as R.

Interpretation of standardised normalised total score and cut-off values

Based on normalised T-scores, we recommend a cut-off value of T ≤ 44 for a challenged PDR, 45 ≤ T ≤ 56 for an average PDR, and T > 56 for a good PDR. A Table with cut-off values for both raw scores and T-scores is provided here.

Raw score	T-score	Interpretation
RS < 23	T < 30	severely challenged
23 ≤ RS ≤ 30	30 ≤ T ≤ 39	moderately challenged
31 ≤ RS ≤ 34	40 ≤ T ≤ 44	mildly challenged
35 ≤ RS ≤ 42	45≤ T ≤ 56	average
42 < RS	56 < T	good

Citation for use of the PDRQ-9:

Van der Feltz-Cornelis CM, Van Oppen P, Van Marwijk HW, De Beurs E, Van Dyck R. A patient-doctor relationship questionnaire (PDRQ-9) in primary care: development and psychometric evaluation. Gen Hosp Psychiatry. 2004 Mar-Apr;26(2):115-20. doi: 10.1016/j.genhosppsych.2003.08.010. PMID: 15038928.

Citation for use of cut-off values and standardised normalised score:

Christina M. van der Feltz-Cornelis, Edwin de Beurs. The Patient-Doctor Relationship Questionnaire (PDRQ-9). An overview of 20 years of research and a proposal for normalisation of scores. Systematic review. Front. Health Serv. 2026:6:1754286.

© Prof.dr. Christina van der Feltz-Cornelis Email: christina.vanderfeltz-cornelis@york.ac.uk 2026

PDRQ-9 – Dutch version

Instructie:

Hieronder staan negen uitspraken die een persoon kan doen over zijn/haar dokter. Geef voor iedere bewering aan in hoeverre deze voor u van toepassing is. U kunt dit doen door 1 van de 5 cijfers te omcirkelen. Het is van belang dat u één getal (antwoordmogelijkheid) per vraag omcirkelt. De nummers betekenen:

1= helemaal niet van toepassing
2= een beetje van toepassing
3= nogal van toepassing
4= in grote mate van toepassing
5= helemaal van toepassing

1.	Mijn dokter is mij behulpzaam	1	2	3	4	5
2.	Mijn dokter heeft voldoende tijd voor mij	1	2	3	4	5
3.	Ik heb vertrouwen in mijn dokter	1	2	3	4	5
4.	Mijn dokter begrijpt mij	1	2	3	4	5
5.	Mijn dokter zet zich voor mij in	1	2	3	4	5
6.	Mijn dokter en ik zijn het eens over de aard van mijn klachten	1	2	3	4	5
7.	Ik kan met mijn dokter praten	1	2	3	4	5
8.	Ik ben tevreden over de behandeling door mijn dokter	1	2	3	4	5
9.	Mijn dokter is goed te bereiken	1	2	3	4	5
	Subtotaal score per antwoordmogelijkheid (alle 1s, alle 2s etc.)					
	Totaalscore alle items samen					

Scoren

Items scoren

De vragenlijst bestaat uit negen items die één enkel onderliggend construct beoordelen: de ervaring van de patiënt met zijn arts. Elk item wordt gescoord op een vijfpuntsantwoordschaal, variërend van 1 tot 5. Alle items worden in dezelfde richting gescoord; hogere itemscores duiden op een positievere ervaring.

Omgaan met ontbrekende antwoorden op items

Als er niet meer dan twee items ontbreken (d.w.z. ≤ 22% van de items), kan de totaalscore worden berekend door de ontbrekende item(s) te vervangen door het gemiddelde van de beantwoorde items voor die respondent. Als er meer dan twee items ontbreken, mogen de totale en gemiddelde scores niet worden berekend en moeten deze als ontbrekend worden behandeld.

Totaalscore

Door de scores van alle 9 items bij elkaar op te tellen, wordt een totaalscore verkregen. De totaalscore varieert van 9 tot 45. Dit is de ruwe score (RS).

Interpretatie van ruwe totaalscores en grenswaarden

Hogere scores weerspiegelen een betere beoordeling van de patiënt-dokter relatie. Voor deze ruwe score adviseren wij grenswaarden aan te houden van < 35 voor een uitdaging op het gebied van de patiënt-dokter relatie, 35 ≤ score ≤ 42 voor een gemiddelde score, en > 42 voor een score passend bij een goede patiënt-dokter relatie.

Standaardisering van ruwe score

De gestandaardiseerde totaal score (T score) wordt verkregen na het normaliseren van de ruwe score (RS) met een polynomiale transformatie. T-scores hebben een gemiddelde (M) van 50 en standaard deviatie (SD) van 10. De gestandaardiseerde, genormaliseerde score kan handmatig berekend worden uit de Ruwe Score (RS) volgens onderstaande formule.

$T_{genormaliseerd} = 6{,}1e\text{-}01+1{,}89*\mathbf{RS}-3{,}865e\text{-}02*\mathbf{RS}^2+6{,}151e\text{-}04*\mathbf{RS}^3.$

Voorbeeld:

Voor een RS van 37 (de gemiddelde totaalscore) is $T_{genormaliseerd}$:

$6{,}1e\text{-}01+1{,}89*\mathbf{37}-3{,}865e\text{-}02*(\mathbf{37}^2)+6{,}151e\text{-}04*(\mathbf{37}^3) =$

$6{,}1e\text{-}01+1{,}89*\mathbf{37}-3{,}865e\text{-}02*1369+6{,}151e\text{-}04*50653 =$

70,54 – 52,91 +31,15 = 48,78, hetgeen T = 50 benadert.

Interpretatie van genormaliseerde totaalscore en grenswaarden

Voor deze genormaliseerde totaalscore adviseren wij grenswaarden aan te houden van ≤ 44 voor een uitdaging op het gebied van de patiënt-dokter relatie, 45 ≤ score ≤ 56 voor een gemiddelde score, en > 56 voor een score passend bij een goede patiënt-dokter relatie.

Formule voor syntax

De volgende formule kan worden ingebouwd in Microsoft Office Excel of andere statistische of computersoftware, zoals R, en levert dan de gestandaardiseerde T-score op. Als volgt:

$T_{genormaliseerd} = 6{,}1e\text{-}01+1{,}89*\mathbf{RS}-3.865e\text{-}02*\mathbf{RS}\char`^2+6{,}151e\text{-}04*\mathbf{RS}\char`^3.$

Citeer bij gebruik van de PDRQ-9:

Van der Feltz-Cornelis CM, Van Oppen P, Van Marwijk HW, De Beurs E, Van Dyck R. A patient-doctor relationship questionnaire (PDRQ-9) in primary care: development and psychometric evaluation. Gen Hosp Psychiatry. 2004 Mar-Apr;26(2):115-20. doi: 10.1016/j.genhosppsych.2003.08.01. PMID: 15038928.

Citeer bij toepassing van de grenswaarden en de genormaliseerde score:

Christina M. van der Feltz-Cornelis, Edwin de Beurs. The Patient-Doctor Relationship Questionnaire (PDRQ-9). An overview of 20 years of research and a proposal for normalisation of scores. Systematic review. Front. Health Serv. 2026:6:1754286.

© Prof.dr. Christina van der Feltz-Cornelis Email: christina.vanderfeltz-cornelis@york.ac.uk 2026
